# Supplementary material for: AXL confers intrinsic resistance to osimertinib and advances the emergence of tolerant cells
Source: Nat Commun. 2019 Jan 16;10:259. doi: 10.1038/s41467-018-08074-0 (PMC6335418; doi:10.1038/s41467-018-08074-0)
Supplement: Supplementary file 1 — Supplementary Information [file 41467_2018_8074_MOESM1_ESM.pdf]

AXL confers intrinsic resistance to osimertinib and advances the emergence of tolerant cells

Taniguchi et al.

Supplementary Information

Supplementary Figure 1

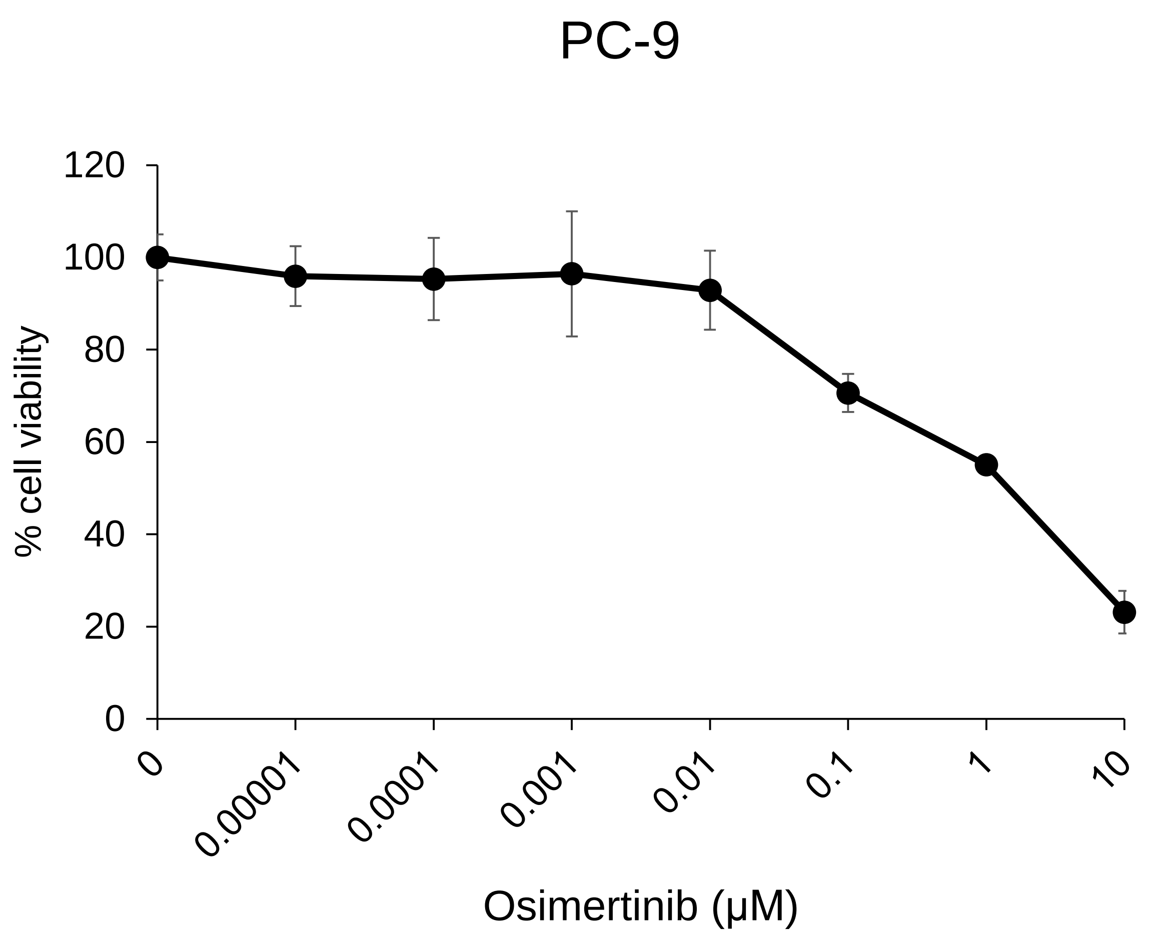

Supplementary Figure 1. Effect of osimertinib on the viability of PC-9 cells *in vitro.*

PC-9 cells were seeded at 2 × 10^3 cells per well in 96-well plates and incubated with osimertinib at the indicated concentrations for 72 h. Cell viability was assessed using MTT assays. Data shown are representative of three independent experiments.

Supplementary Figure 2

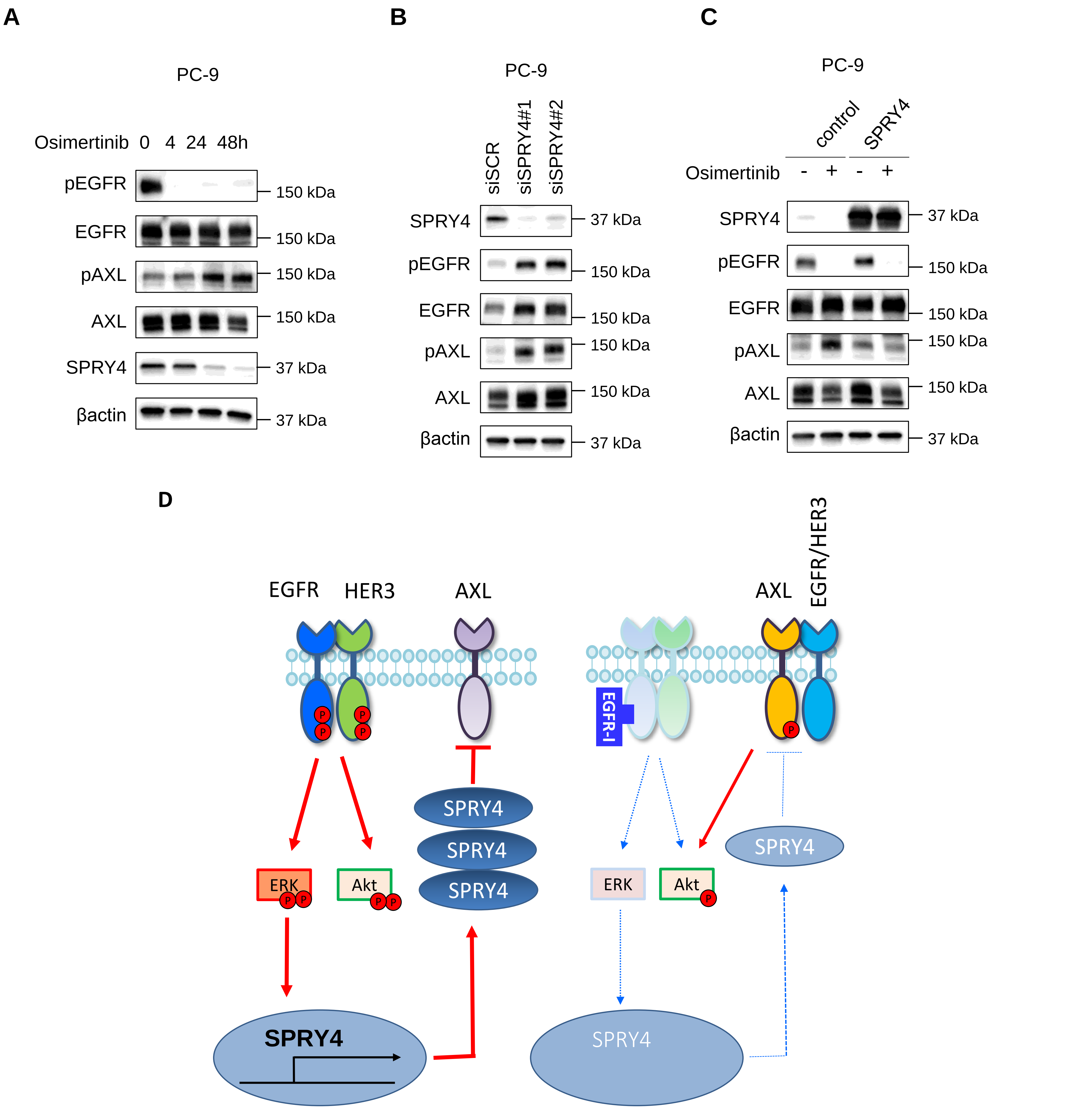

Supplementary Figure 2. SPRY4 was a negative modulator for the activation of the AXL axis in *EGFR*-mutated NSCLC cells treated with osimertinib.

(A) PC-9 cells were treated with osimertinib (100 nmol/L), lysed, and the indicated proteins detected by western blotting. **(B)** PC-9 cells were transfected with two different siRNAs targeting *SPRY4* or scrambled siRNA and cultured for 72 h. Lysates were probed with the indicated antibodies. **(C)** PC-9 cells infected with a vector control or *SPRY4*-expressing lentiviral plasmids were treated with osimertinib for 48 h. Cell lysates were probed with the indicated antibodies. **(D)** Schematic diagram showing the mechanism of osimertinib-induced tolerance due to AXL activation in NSCLC cells harboring *EGFR* mutations.

Supplementary Figure 3

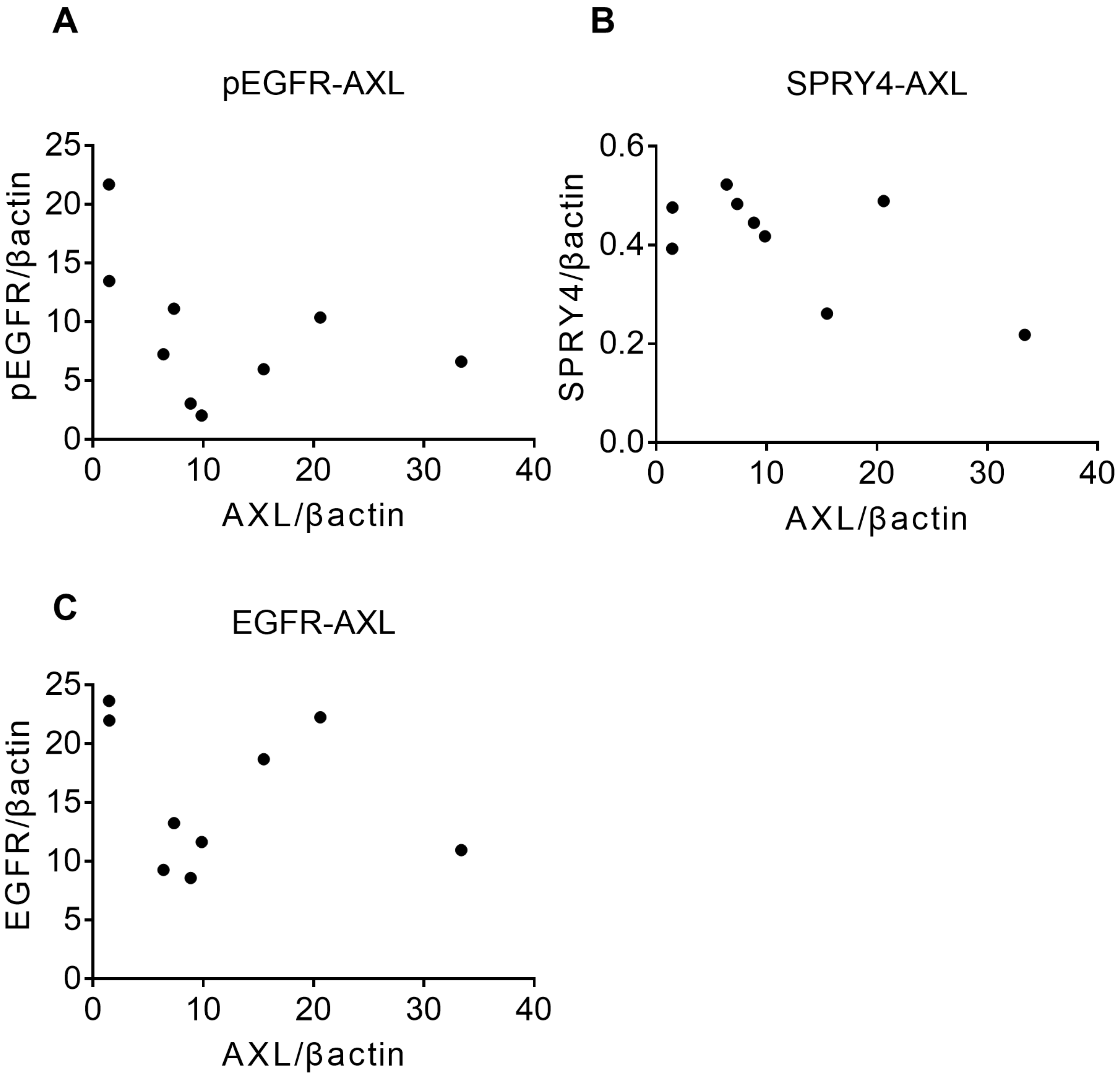

Supplementary Figure 3. Correlation between AXL and the indicated proteins in the lysate of *EGFR*-mutated NSCLC cells

(A) Correlation between the ratio of AXL to actin and the ratio of p-EGFR to βactin (Spearman rank correlation = -0.6; P = 0.048). **(B)** Correlation between the ratio of AXL to βactin and the ratio of SPRY4 to βactin (Spearman rank correlation = -0.644; P = 0.061). **(C)** Correlation between the ratio of AXL to βactin and the ratio of total EGFR to βactin (Spearman rank correlation = -0.233; P = 0.276).

Supplementary Figure 4

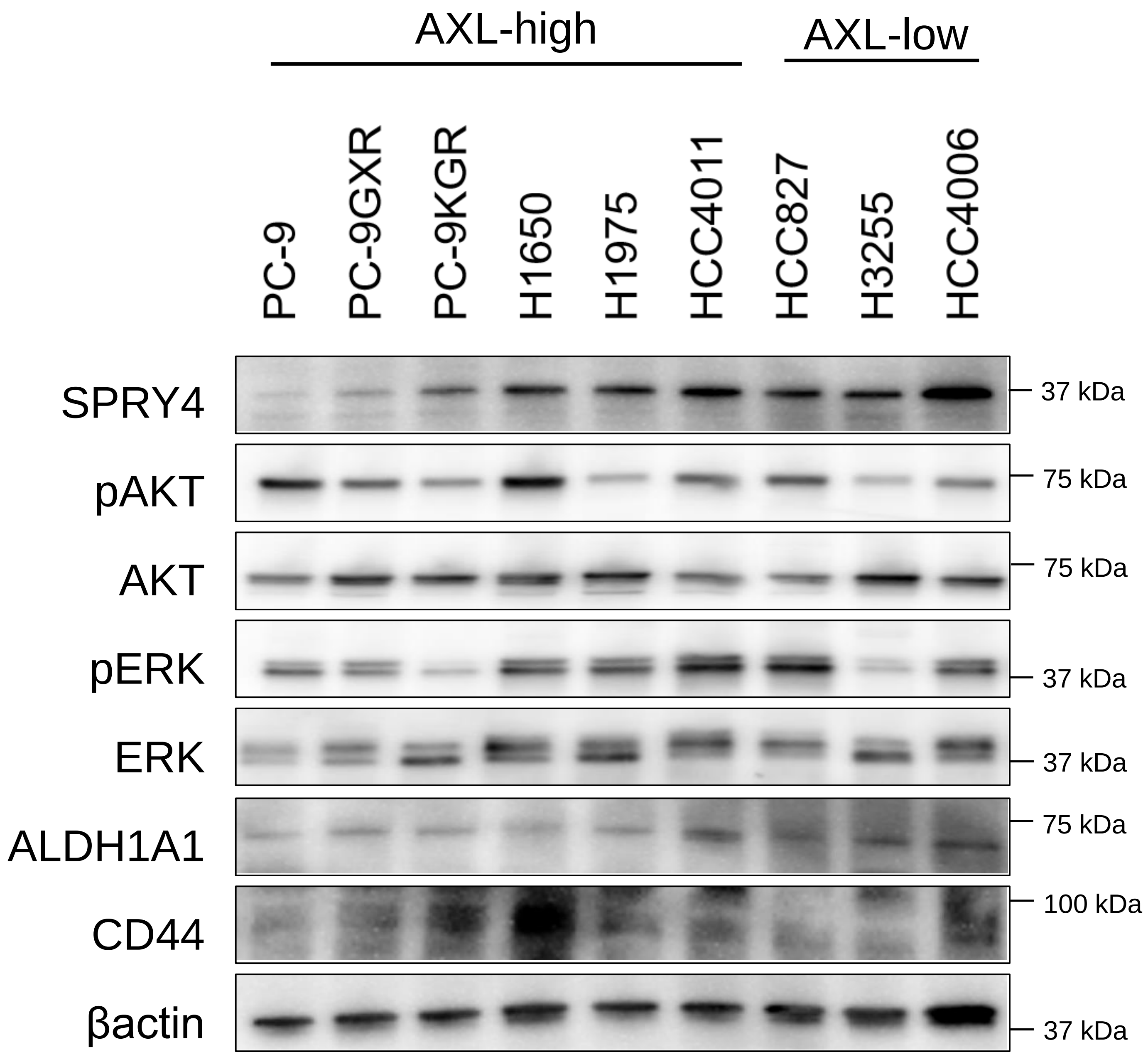

Supplementary Figure 4. Expression of SPRY4 and stem cell-related proteins in *EGFR*-mutated NSCLC cells

The *EGFR*-mutated NSCLC cell lines PC-9, PC-9GXR, PC-9KGR, H1650, H1975, HCC4011, HCC827, H3255, and HCC4006 were lysed, and the indicated proteins were detected by western blotting.

Supplementary Figure 5

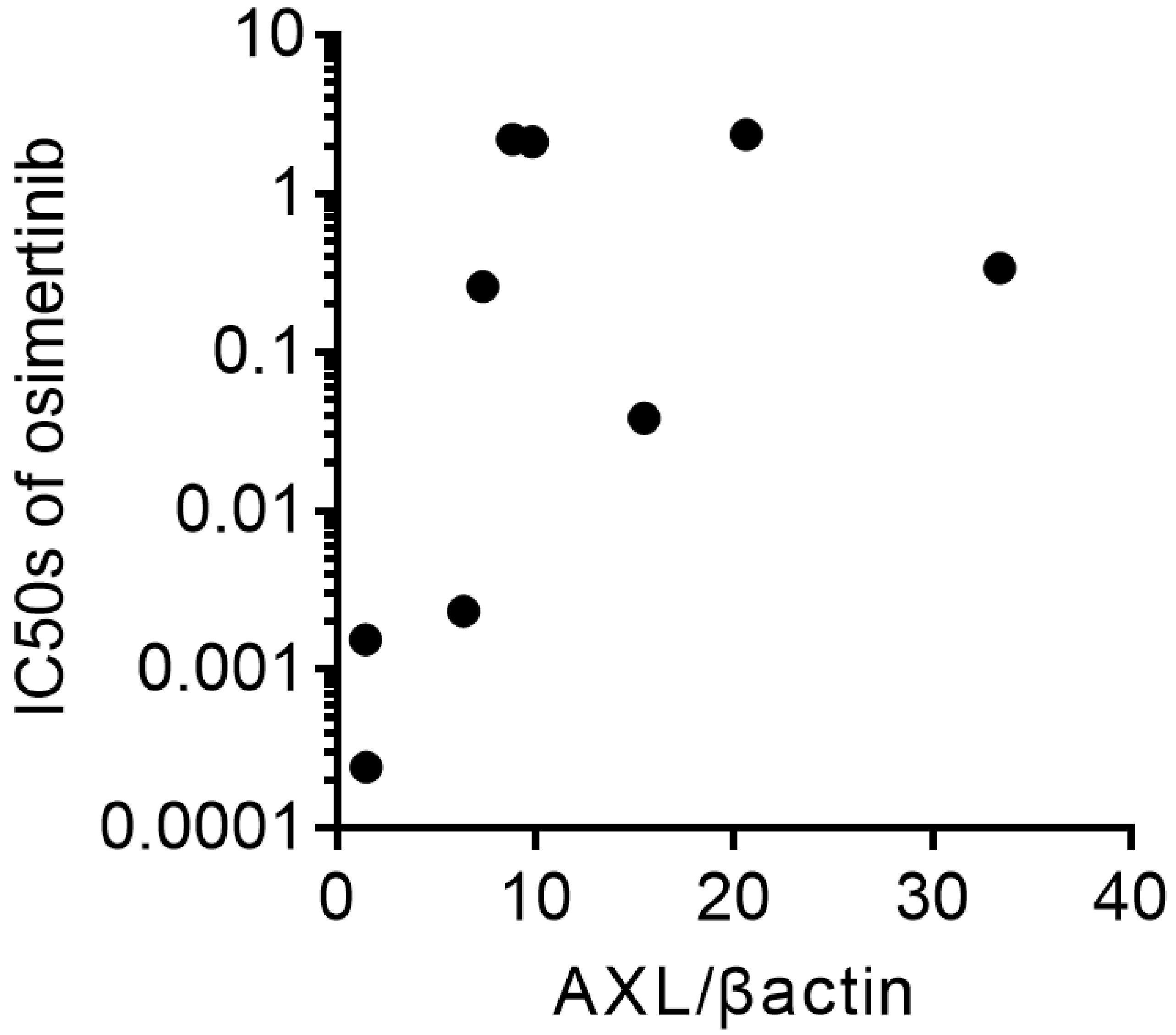

Supplementary Figure 5. Correlation between the ratio of AXL to βactin and the IC50 values for osimertinib in *EGFR*-mutated NSCLC cells.

Spearman rank correlations = 0.733; P = 0.031.

Supplementary Figure 6

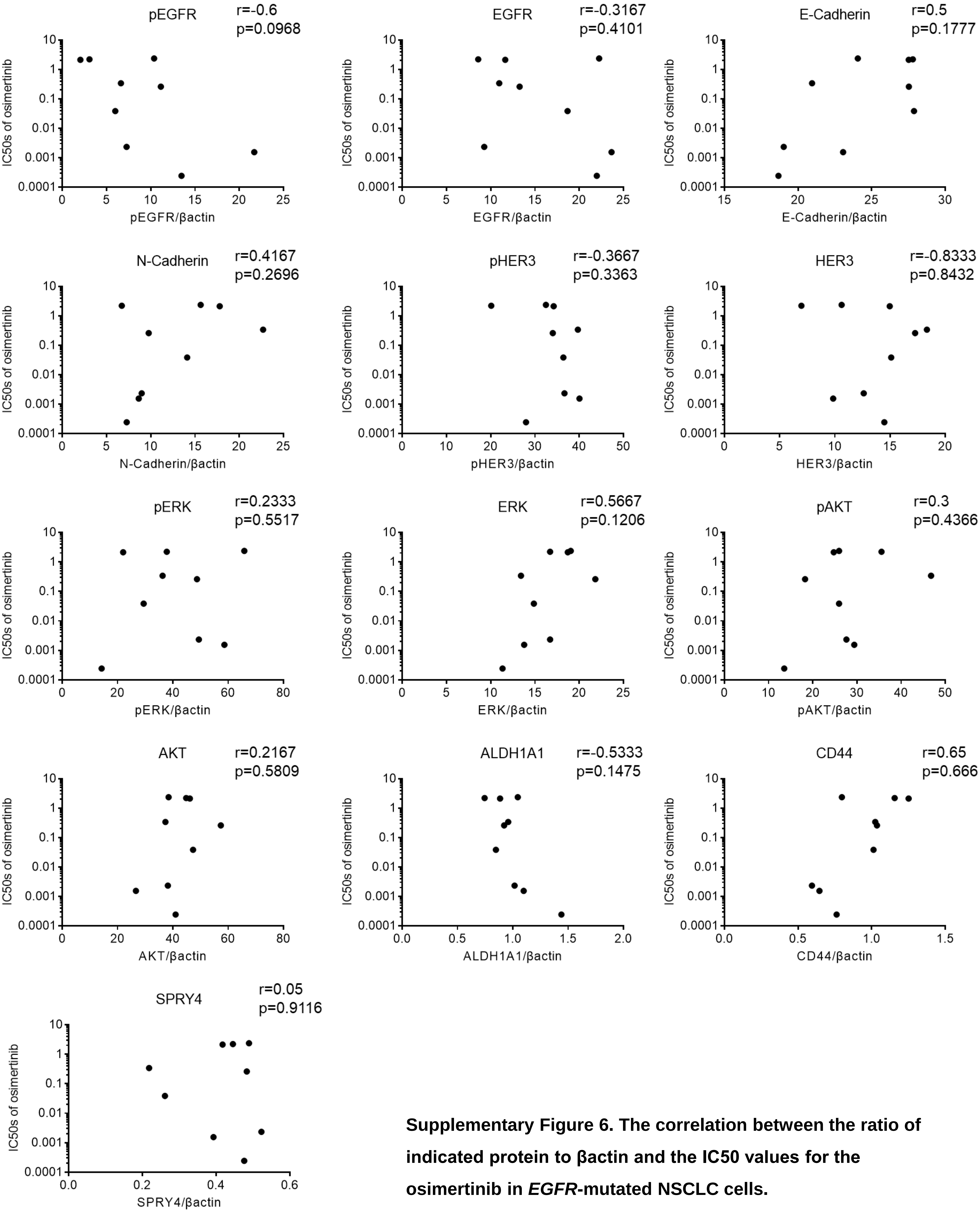

Supplementary Figure 6. The correlation between the ratio of indicated protein to βactin and the IC50 values for the osimertinib in *EGFR*-mutated NSCLC cells.

Supplementary Figure 7

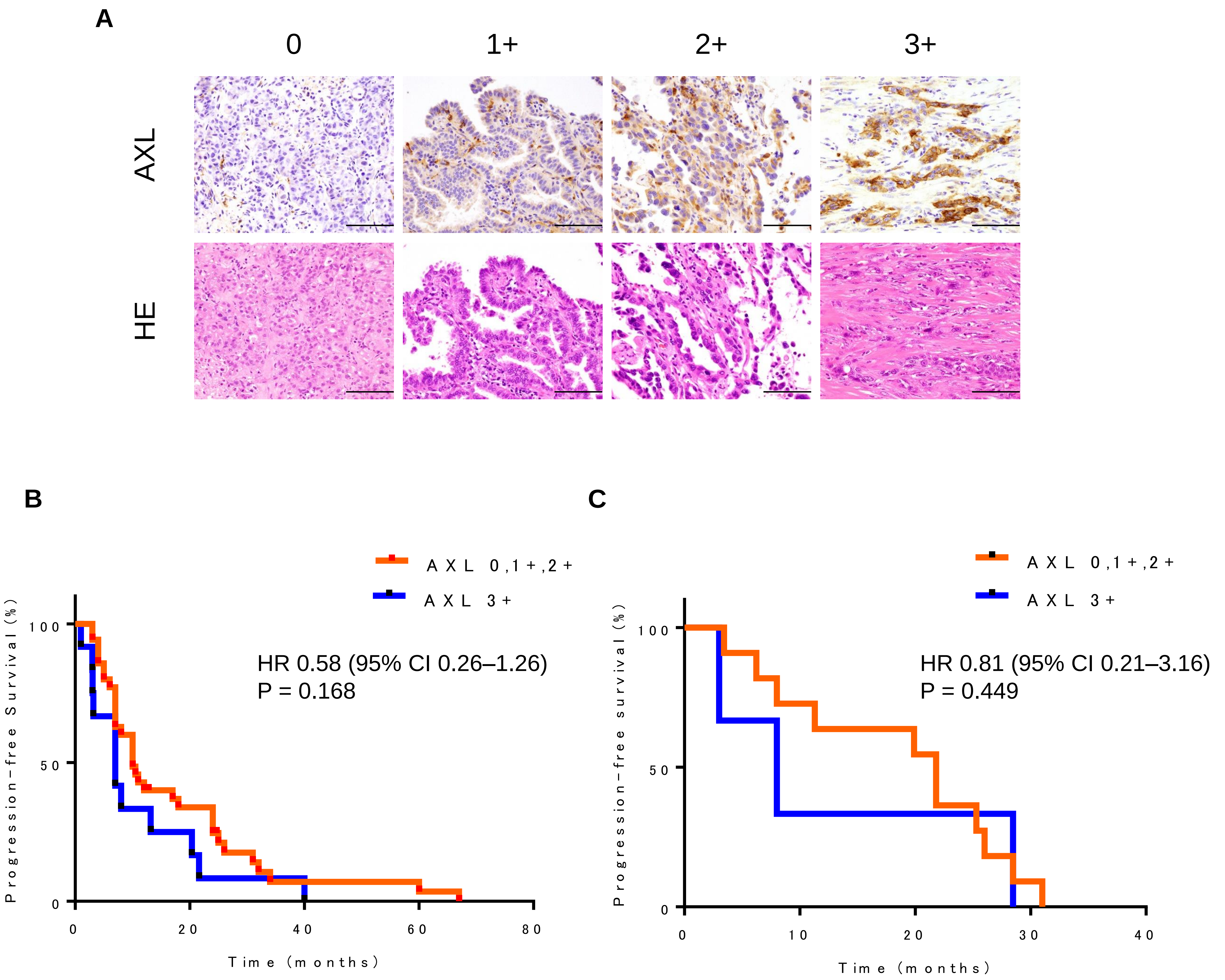

Supplementary Figure 7. Clinical significance of high AXL protein expression on EGFR-TKI treatment in patients with *EGFR*-mutated NSCLC.

(A) Representative results of immunohistochemical staining for AXL expression. Bar, 100 µm. **(B, C)** Kaplan-Meier analysis of the progression-free survival for the first- and second generation EGFR-TKIs **(B)** and osimertinib **(C)** in patients with *EGFR*-mutated NSCLC tumors expressing high (3+; blue line) and low (0, 1+, 2+; red line) levels of AXL protein. A hazard ratio (HR) < 1 implies a low risk of progression for the low-AXL-expression group. **(B)** HR 0.58 (95% CI 0.26–1.26, P = 0.168) and **(C)** HR 0.81 (95% CI 0.21–3.16, P = 0.449).

Supplementary Figure 8

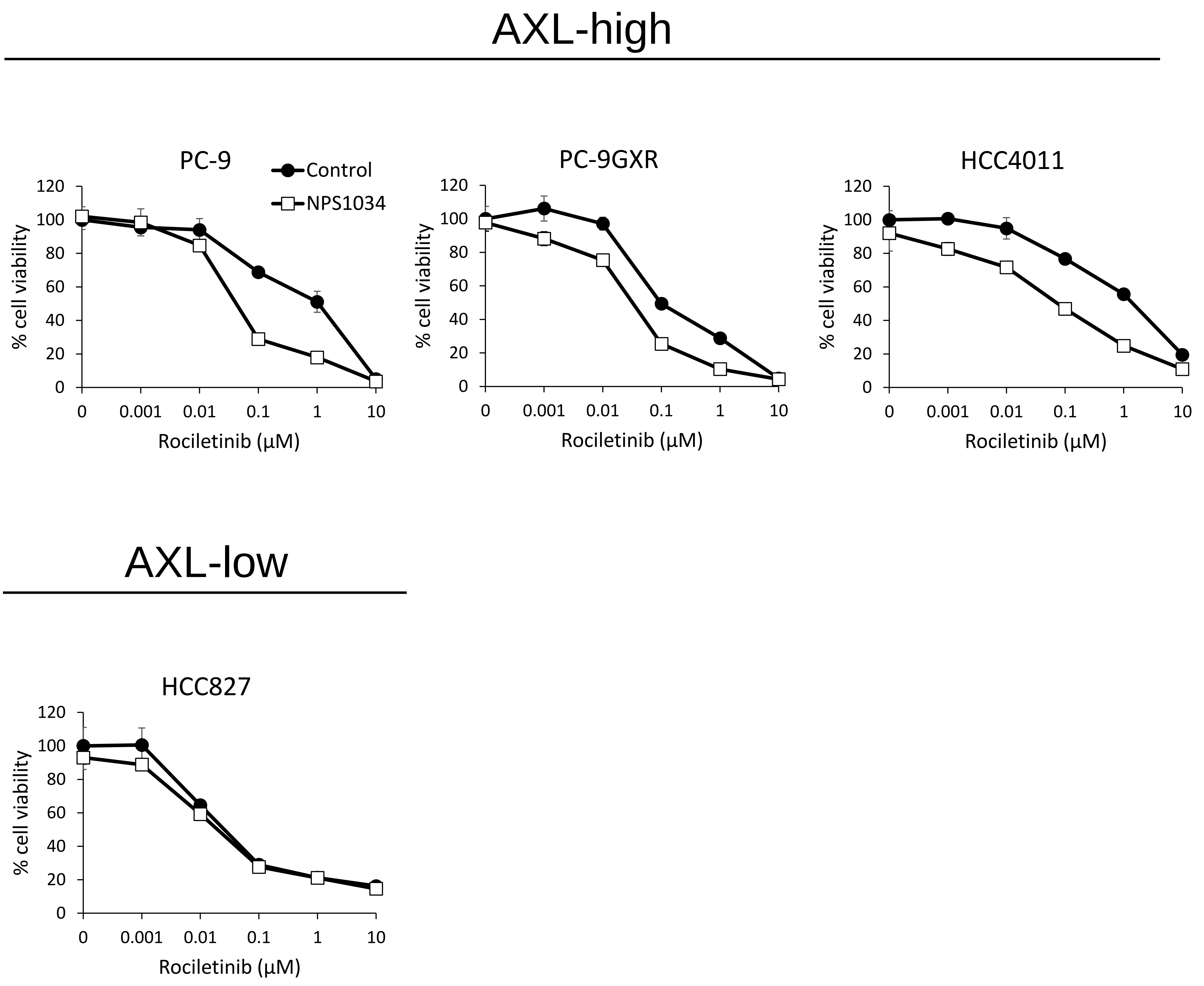

Supplementary Figure 8. Effect of treatment with rociletinib plus NPS1034 on the viability of *EGFR*-mutated NSCLC cells

PC-9, PC-9GXR, HCC4011, and HCC827 cells were incubated with rociletinib in the presence or absence of the AXL inhibitor NPS1034 (1 µmol/L) for 72 h, and cell viability was determined using the MTT assay. Data are representative of three independent experiments that produced similar results.

Supplementary Figure 9

A

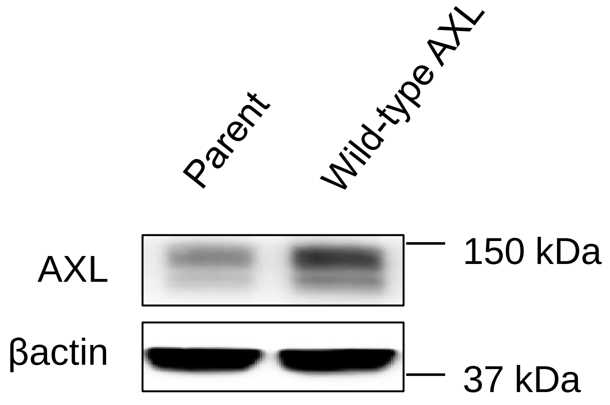

B

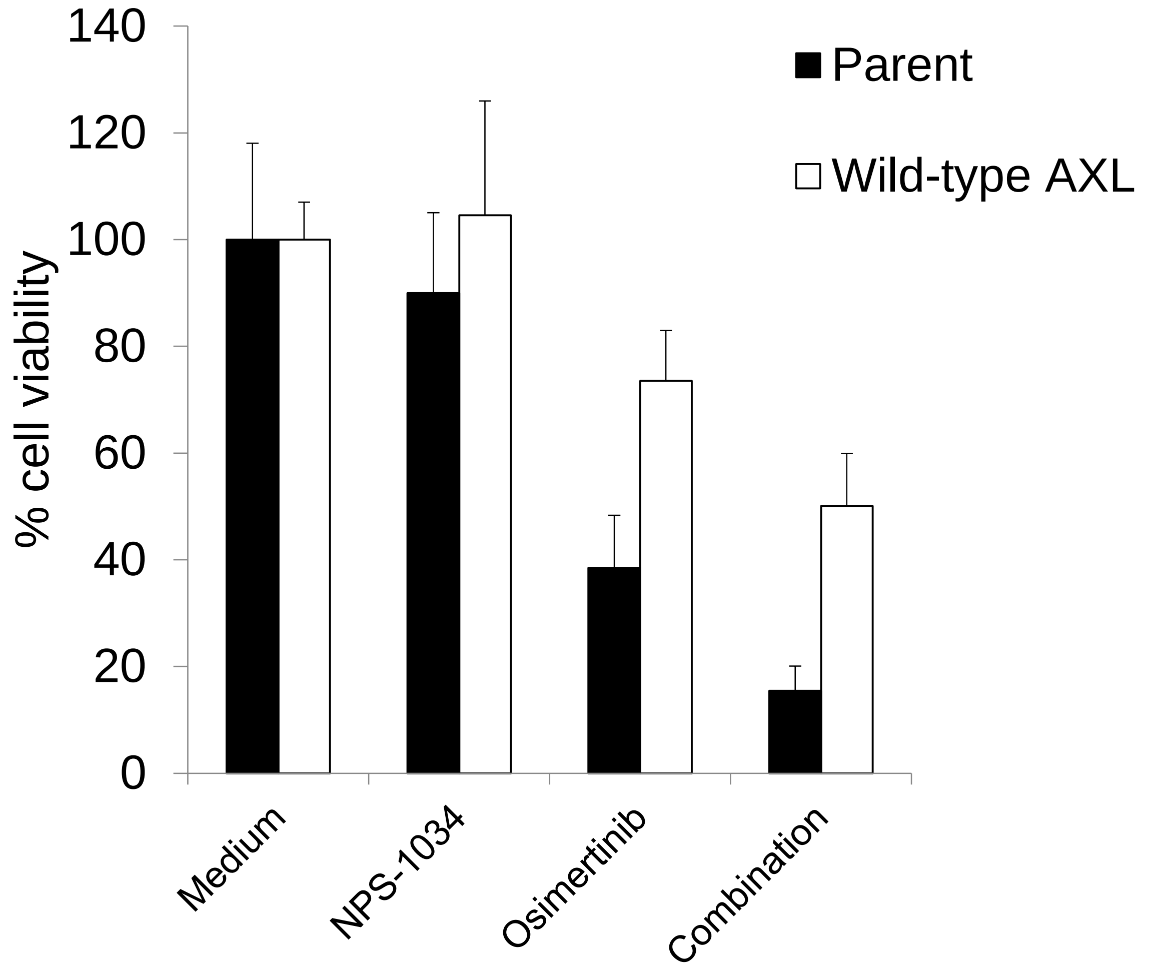

Supplementary Figure 9. Effect of wild-type AXL overexpression in PC-9 cells treated with osimertinib and NPS1034.

(A) Transfection of wild-type AXL resulted in increased expression of AXL protein in PC-9 cells. **(B)** The effect of wild-type AXL overexpression in PC-9 cells were incubated with osimertinib (3 μmol/L) and NPS1034 (1 μmol/L) for 72 h and cell viability was determined using the MTT assay. Data are representative of two independent experiments that produced similar results.

Supplementary Figure 10

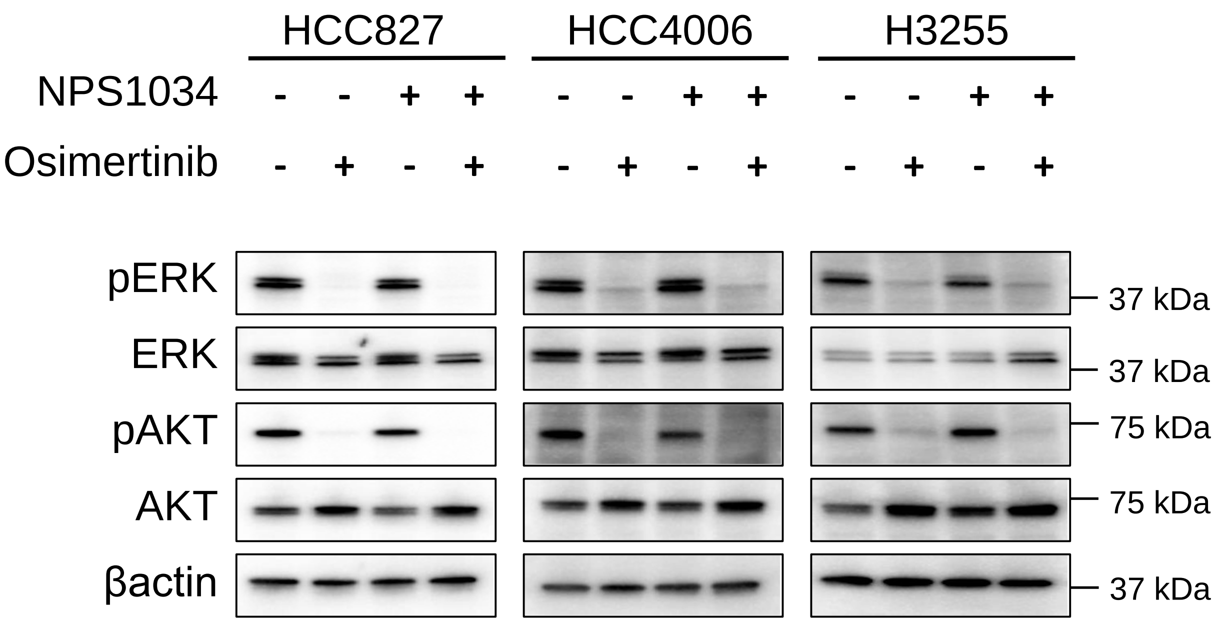

Supplementary Figure 10. Effect of NPS1034 in combination with osimertinib in *EGFR*-mutated NSCLC cells with low AXL protein levels

The indicated cells of the AXL low group were incubated with osimertinib (100 nmol/L), with or without NPS1034 (1 μmol/L), for 1 h. The cells were lysed, and the indicated proteins were detected by western blotting.

Supplementary Figure 11

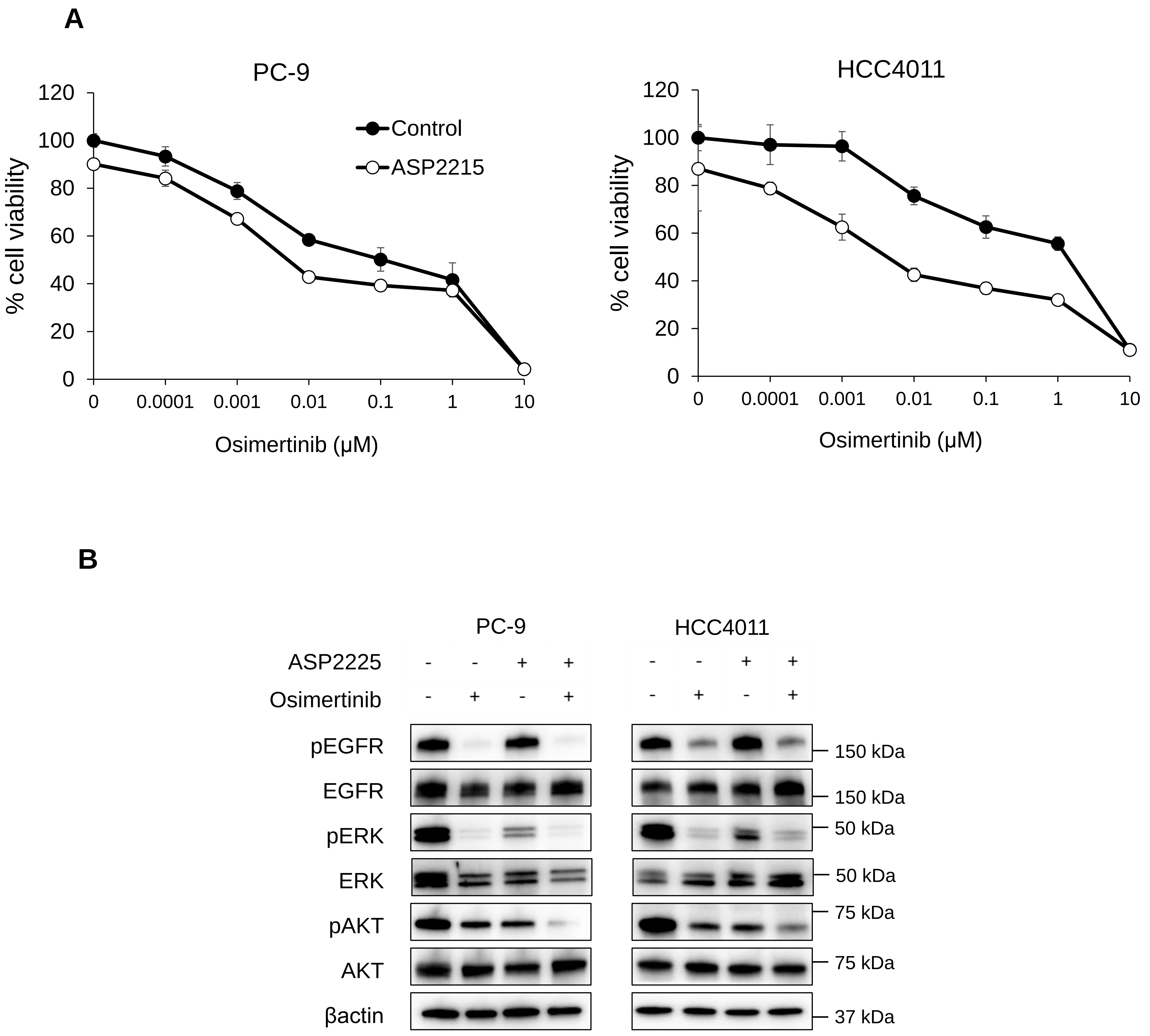

Supplementary Figure 11. Effect of treatment with osimertinib plus ASP2215 on the viability and signal transduction of *EGFR*-mutated NSCLC cells.

(A) *EGFR*-mutated NSCLC cells (PC-9 and HCC4011) were seeded at 2 × 10^3 cells per well in 96-well plates and were incubated with the indicated concentrations of osimertinib with or without ASP2215 (100 nmol/L) for three days. Viability was assessed using MTT assays. Data are representative of three independent experiments. **(B)** PC-9 and HCC4011 cells were treated with osimertinib (100 nmol/L) with or without ASP2215 (100 nmol/L) for 1 h. The cells were lysed and the indicated proteins were detected by western blotting.

Supplementary Figure 12

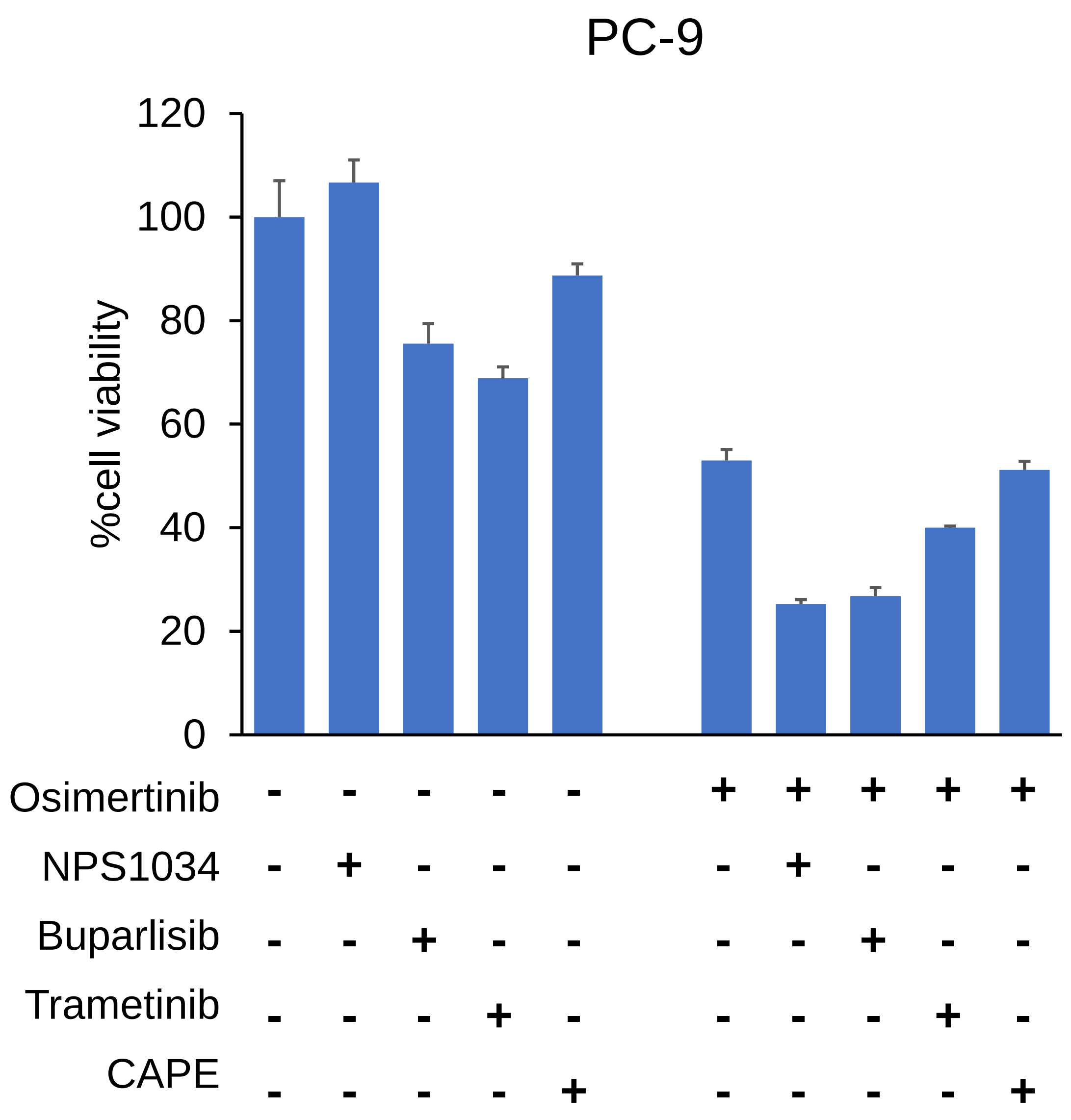

Supplementary Figure 12. Additional effect of treatment with the indicated inhibitors, with or without osimertinib, on the viability of *EGFR*-mutated NSCLC cells

PC-9 cells were incubated with the AXL inhibitor NPS1034 (1 μmol/L), the AKT inhibitor buparlisib (300 nmol/L), the MEK inhibitor trametinib (100 nmol/L), or the NFκB inhibitor CAPE (1 μmol/L), in the presence or absence of osimertinib (100 nmol/L) for 72 h, and cell viability was determined using the MTT assay. Data are representative of three independent experiments that produced similar results.

Supplementary Figure 13

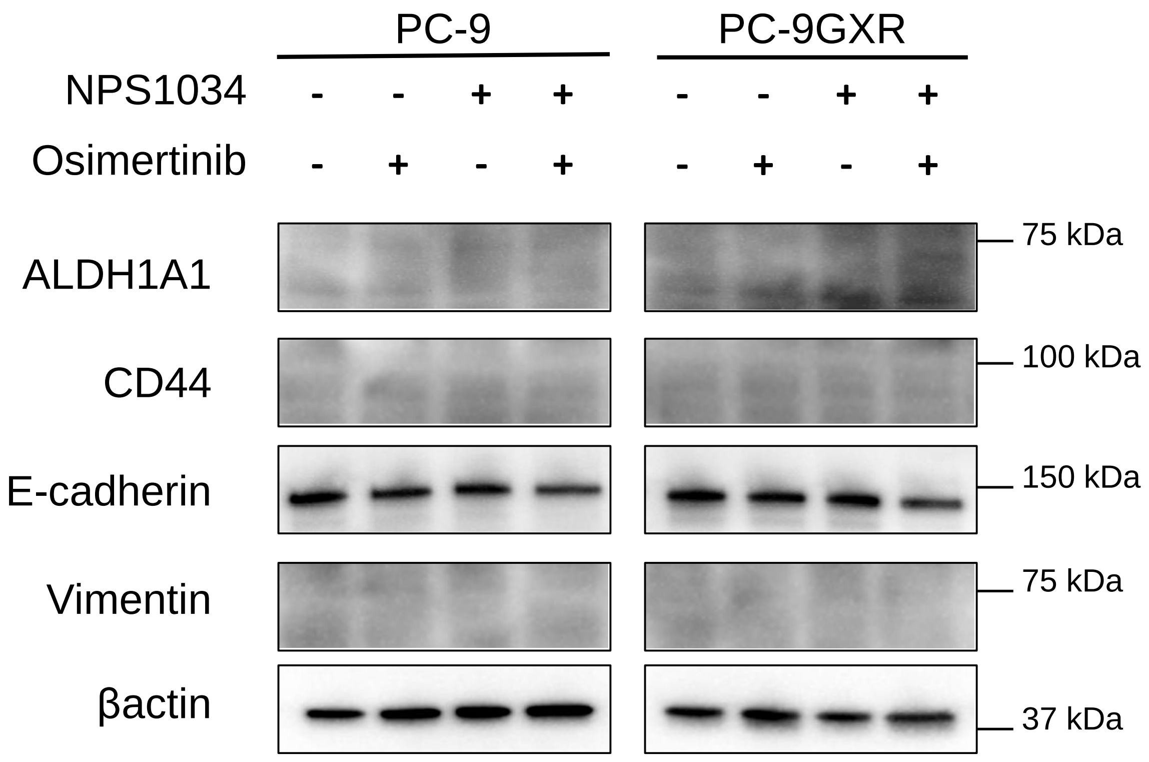

Supplementary Figure 13. Effect of treatment with osimertinib plus NPS1034 on signal transduction of AXL-related proteins in *EGFR*-mutated NSCLC cells.

The indicated cells were incubated with osimertinib (100 nmol/L) with or without NPS1034 (1 μmol/L) for 72 h. The cells were lysed and the indicated proteins were detected by western blotting.

Supplementary Figure 14

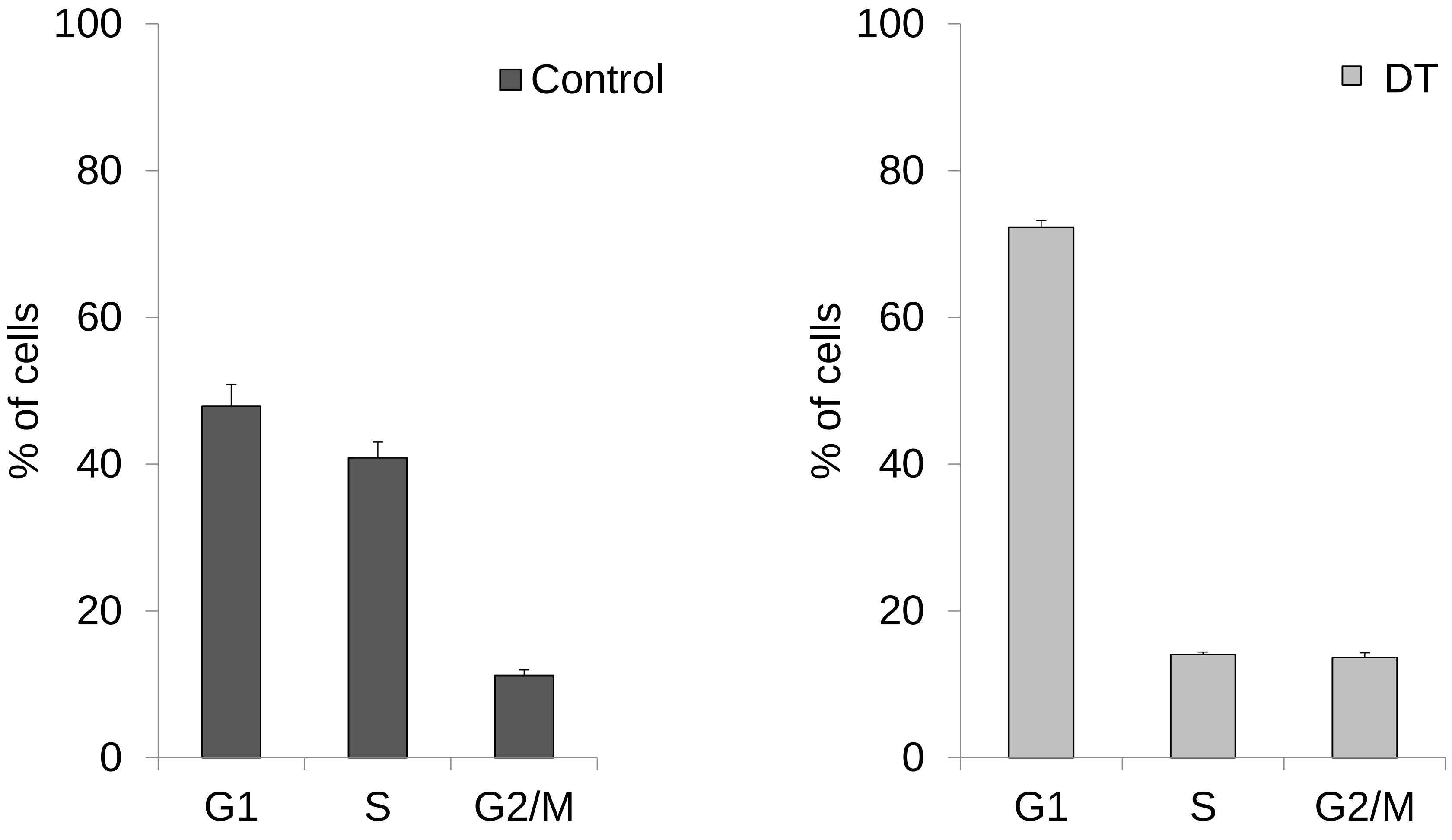

Supplementary Figure 14. Cell cycle analysis in the PC-9 cells.

PC-9 cells were incubated with medium or osimertinib (1 μmol/L) for three days and then the cell cycle was analyzed by flow cytometry using BrdU. The results shown are the percentages of cell populations in the various cell-cycle phases in mock-treated control cells (left) and osimertinib-treated cells (right).

Supplementary Figure 15

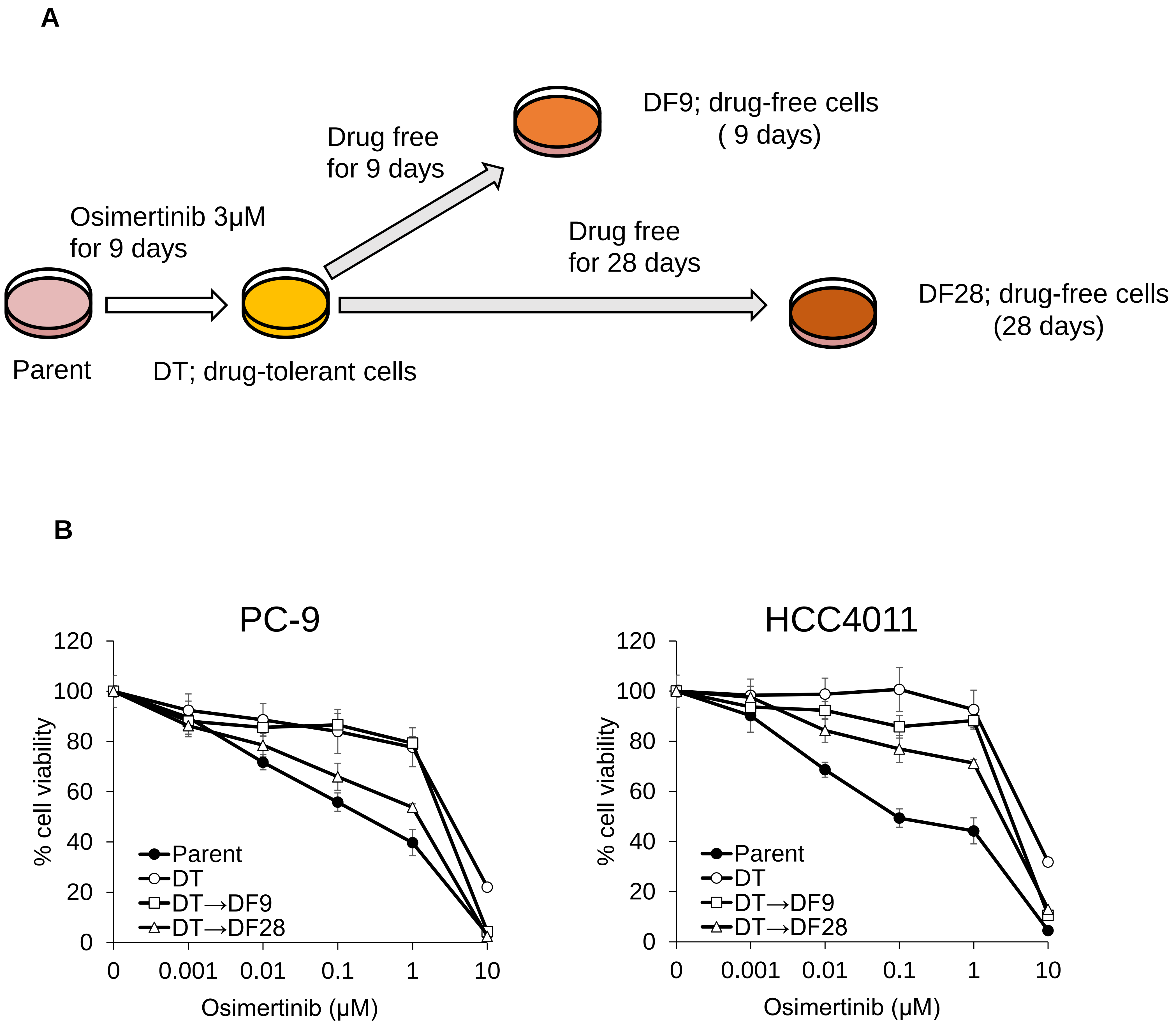

Supplementary Figure 15. AXL-mediated resistance was irreversible even though there was interval of 9 days with drug free.

(A, B) We established the drug-tolerant cells (DT) cells treated with osimertinib 3 μM for 9 days. Then, these cells were incubated without osimertinib for 9 days or 28 days, in which defined as cells with drug-free (DF), which were treated with the indicated concentrations of osimertinib for 72 h and their viability was assessed using MTT assays.

Supplementary Figure 16

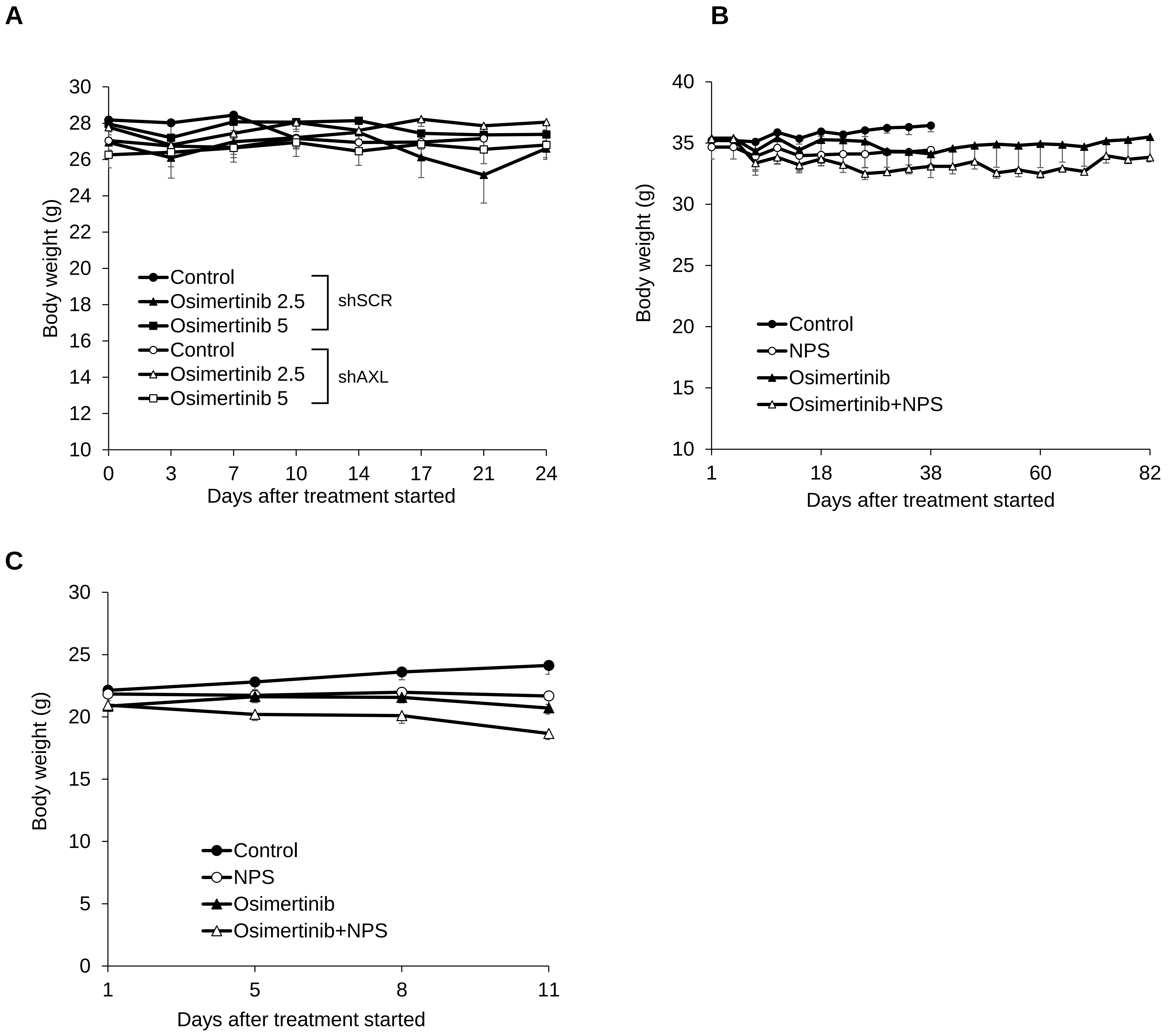

Supplementary Figure 16. The combination of osimertinib and NPS1034 is well tolerated in various xenograft models of *EGFR*-mutated NSCLC.

(A) Stable PC-9 cell-lines were generated by introducing *AXL*-targeting shRNAs and nonspecific control shRNAs. Following subcutaneous injection of the transduced cells into nude mice (n = 5 per group), vehicle (control) or osimertinib (2.5 or 5 mg/kg) were administered five days a week for 24 days. The weights of the mice were evaluated twice weekly. Bars indicate standard errors. **(B)** PC-9 cells were inoculated into the flanks of severe combined immunodeficiency (SCID) mice and the mice (n = 6 per group) were treated with osimertinib (5 mg/kg), NPS1034 (50 mg/kg), or a combination of both osimertinib (5 mg/kg) and NPS1034 (50 mg/kg). The treatments were administered five days a week by oral gavage for 82 days. The weights of the mice were evaluated twice weekly. Bars indicate standard errors. **(C)** PDX tumors (TM00784) were treated with 50 mg/kg NPS1034, 5 mg/kg osimertinib, a combination of both 50 mg/kg NPS1034 and 5 mg/kg osimertinib, and vehicle (control) by daily oral gavage. Mouse weights were evaluated twice weekly. Bars indicate standard error (n = 4 mice per group).

Supplementary Figure 17

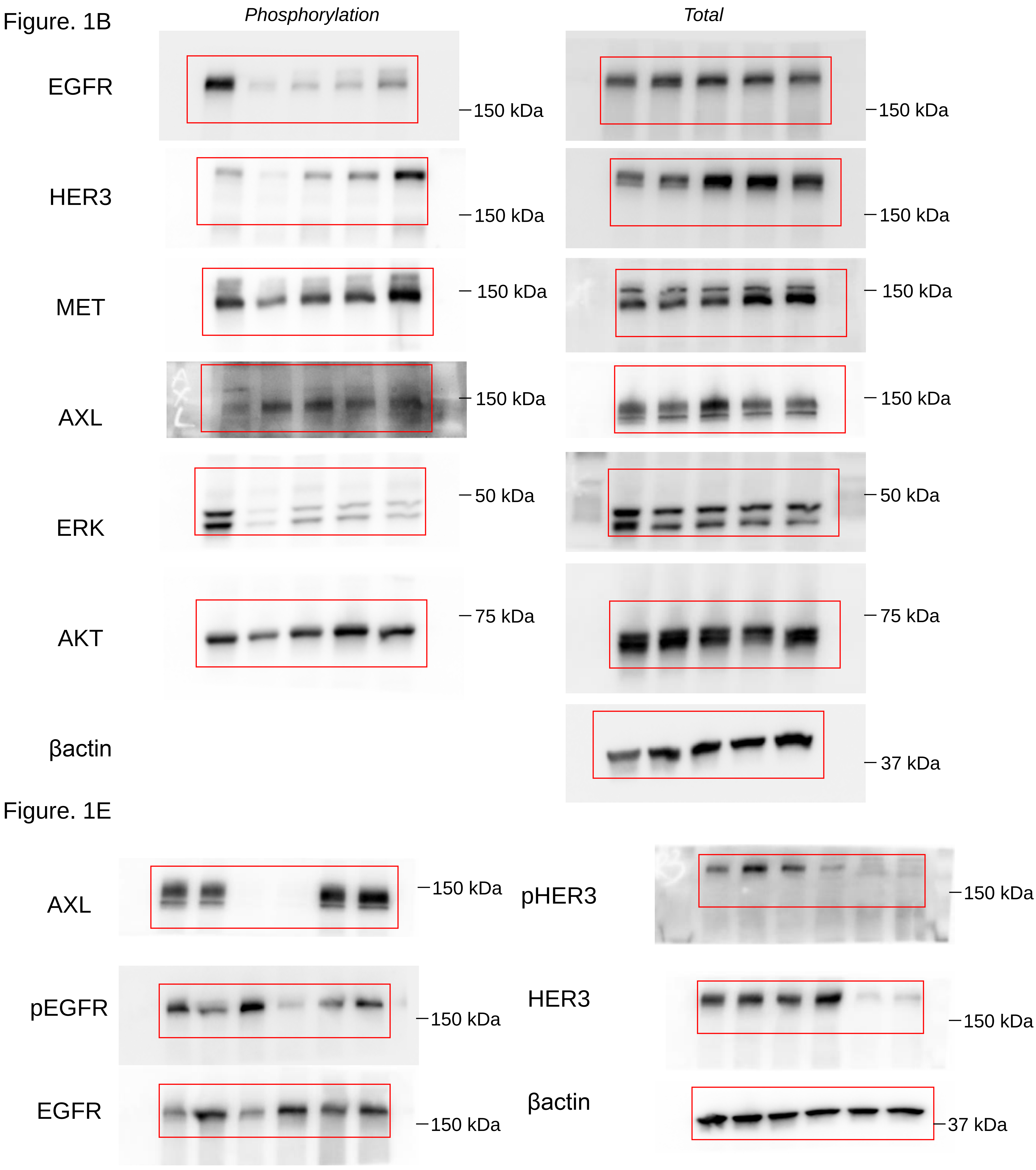

Supplemental Figure 17. Uncropped blots for main figures. Blots shown in the main article are depicted by boxed regions in each of associated uncropped scans

Supplemental Figure 17

Figure. 1F

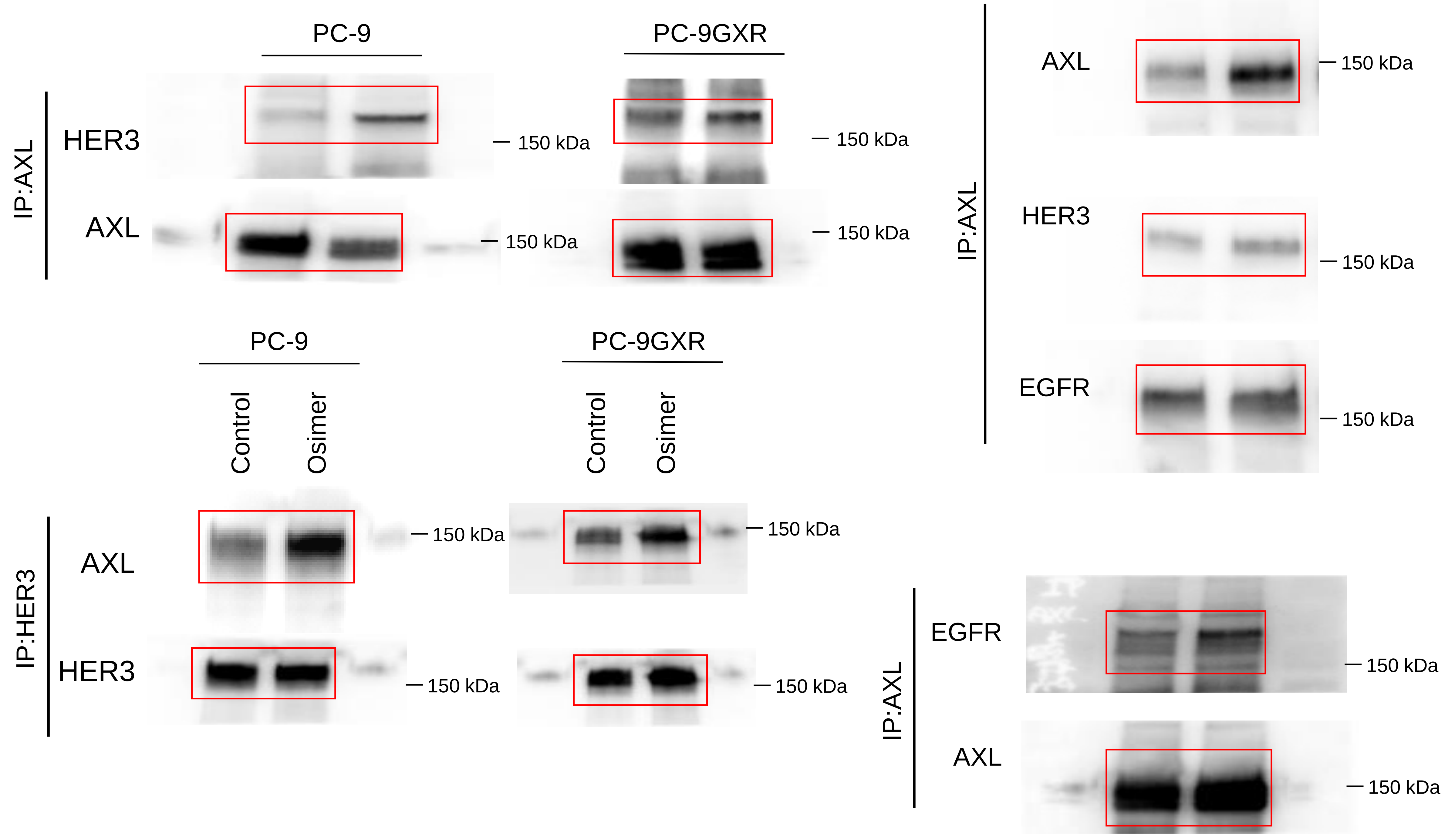

Figure. 2A

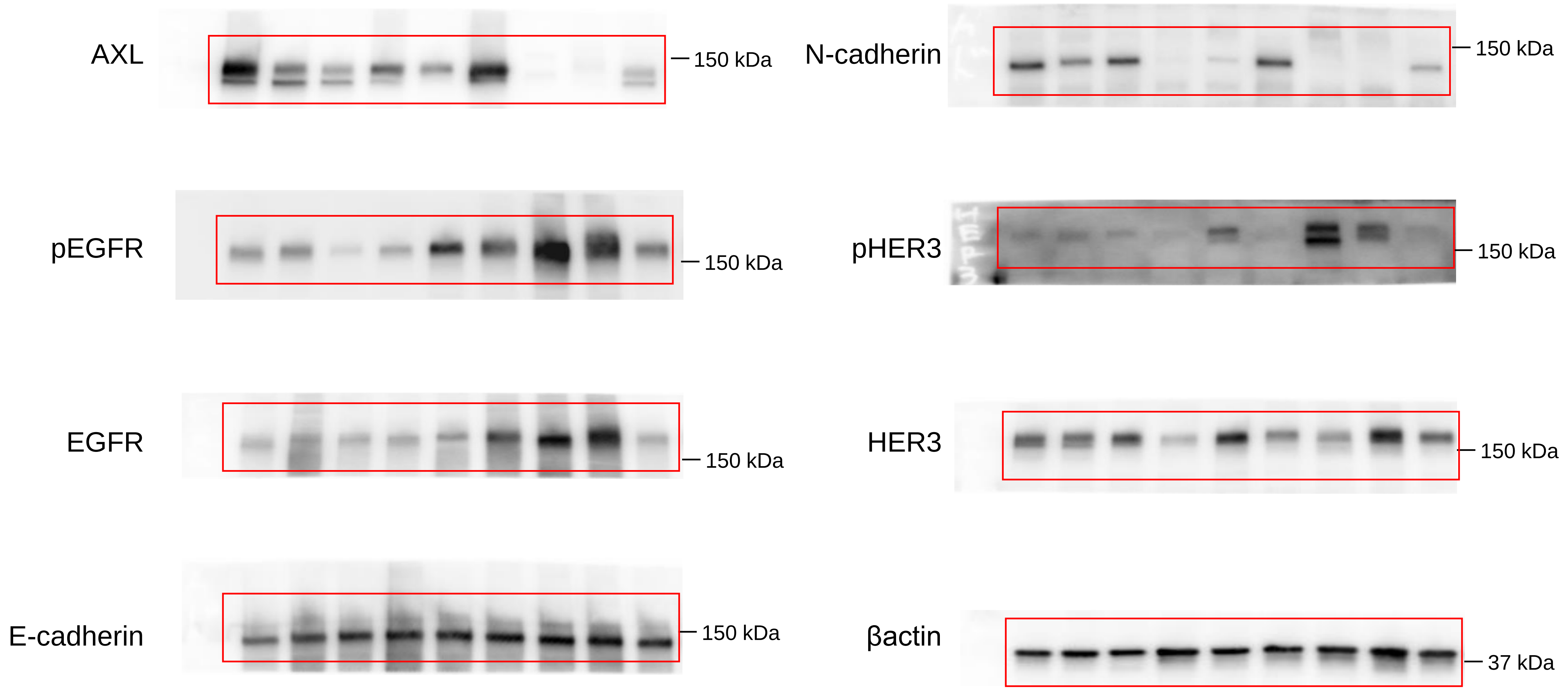

Supplemental Figure 17

Figure. 3C

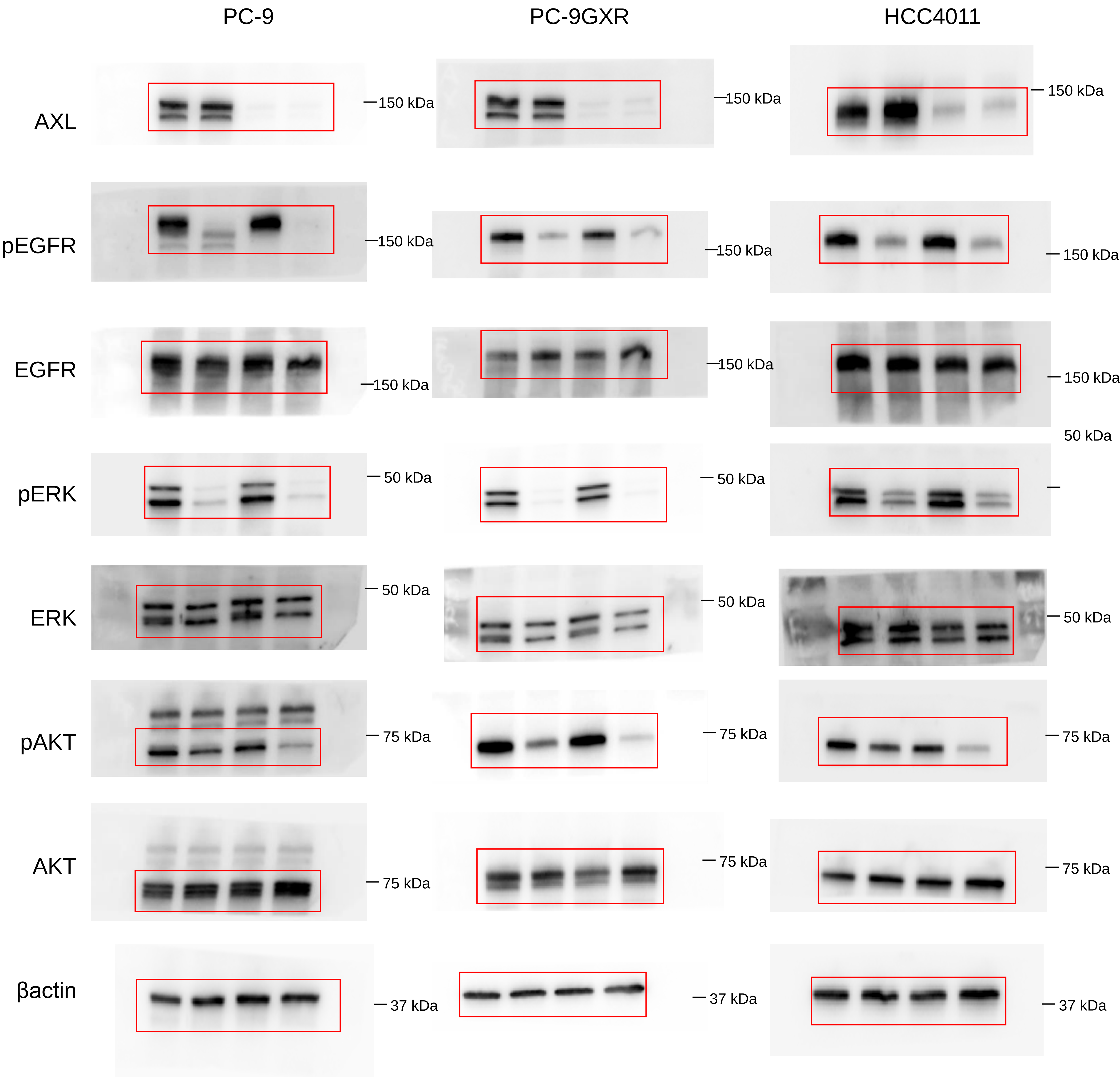

Supplemental Figure 17

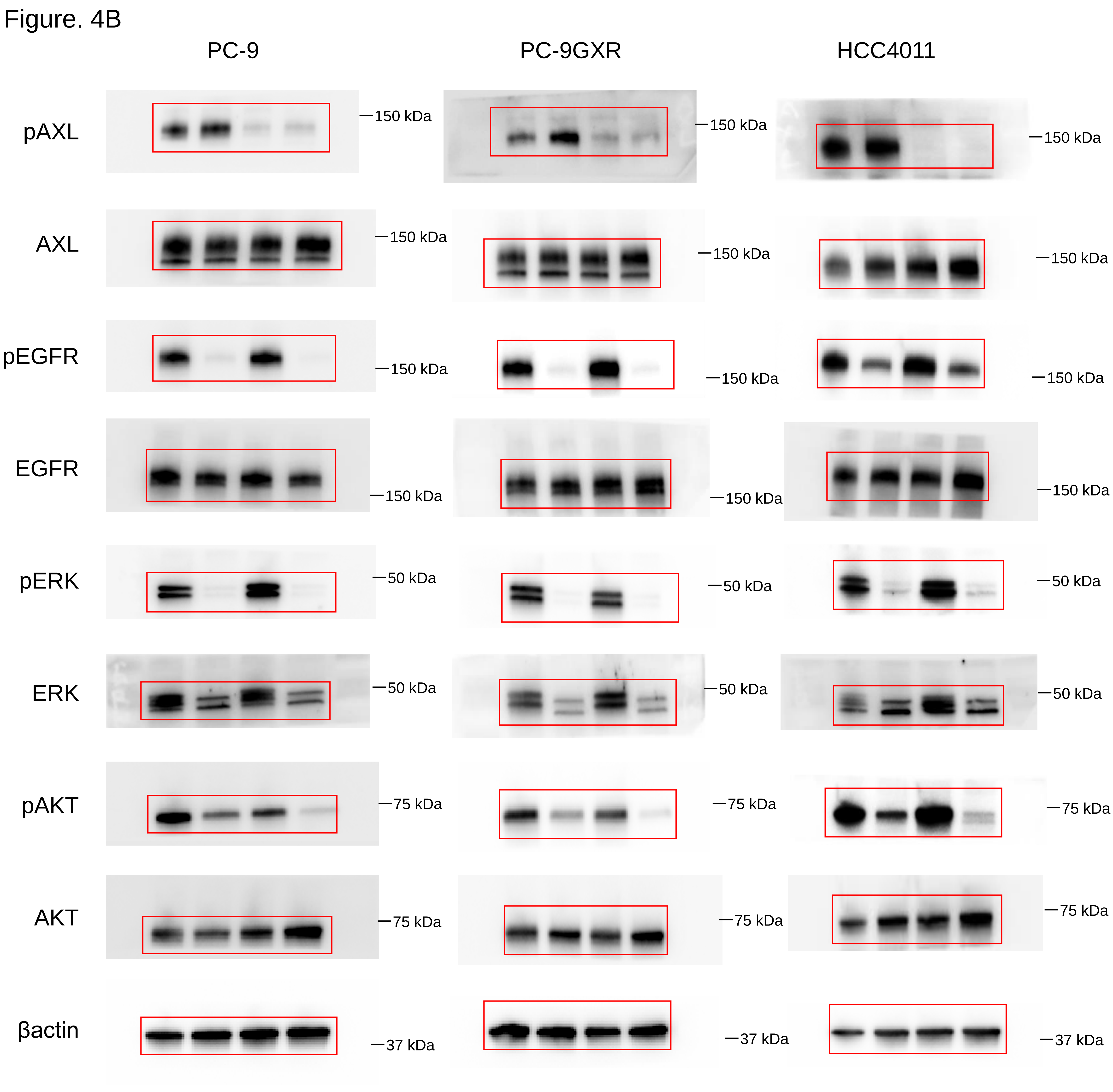

Supplemental Figure 17

Figure. 4C

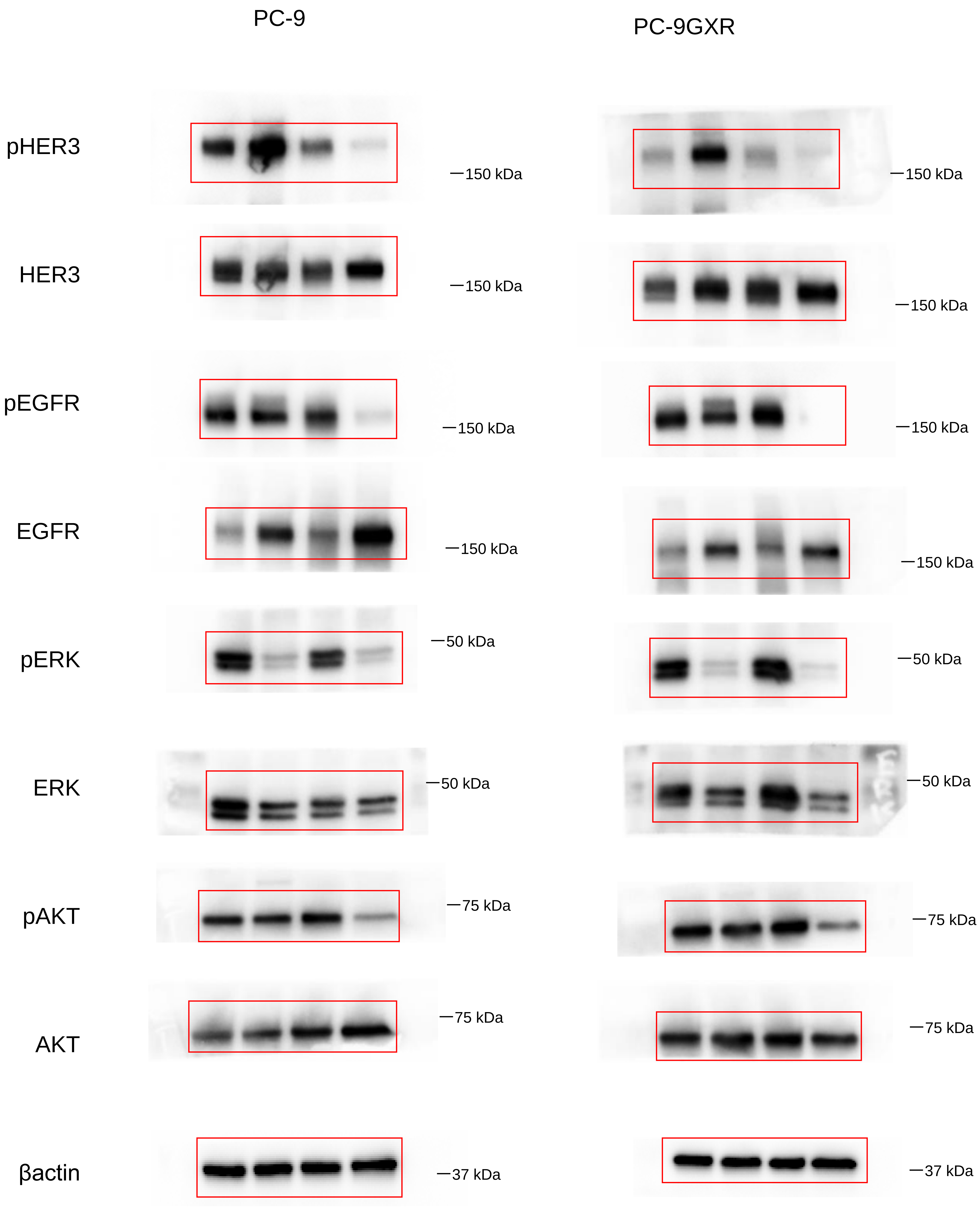

Supplemental Figure 17

Figure. 5B

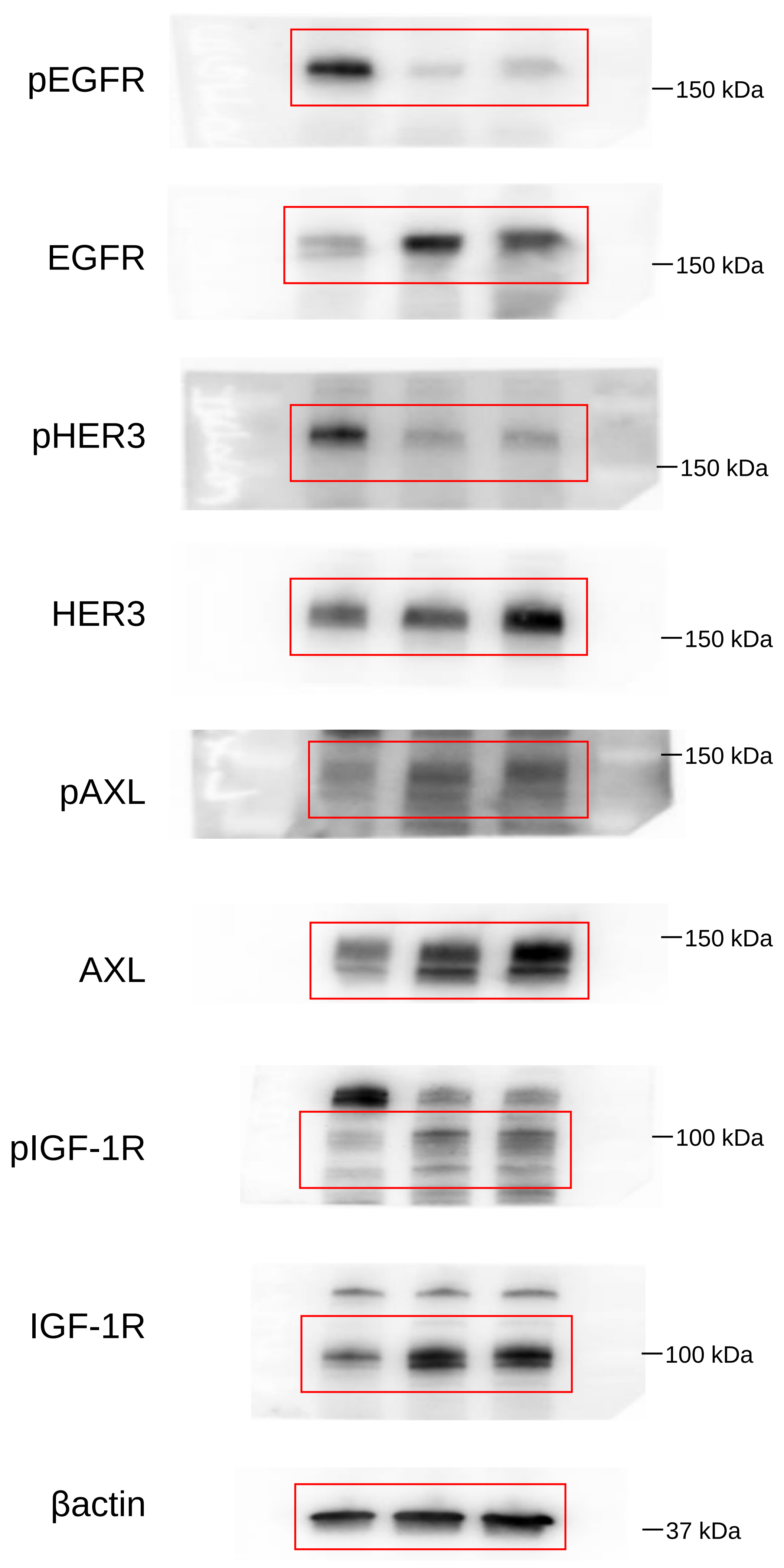

Figure. 5C

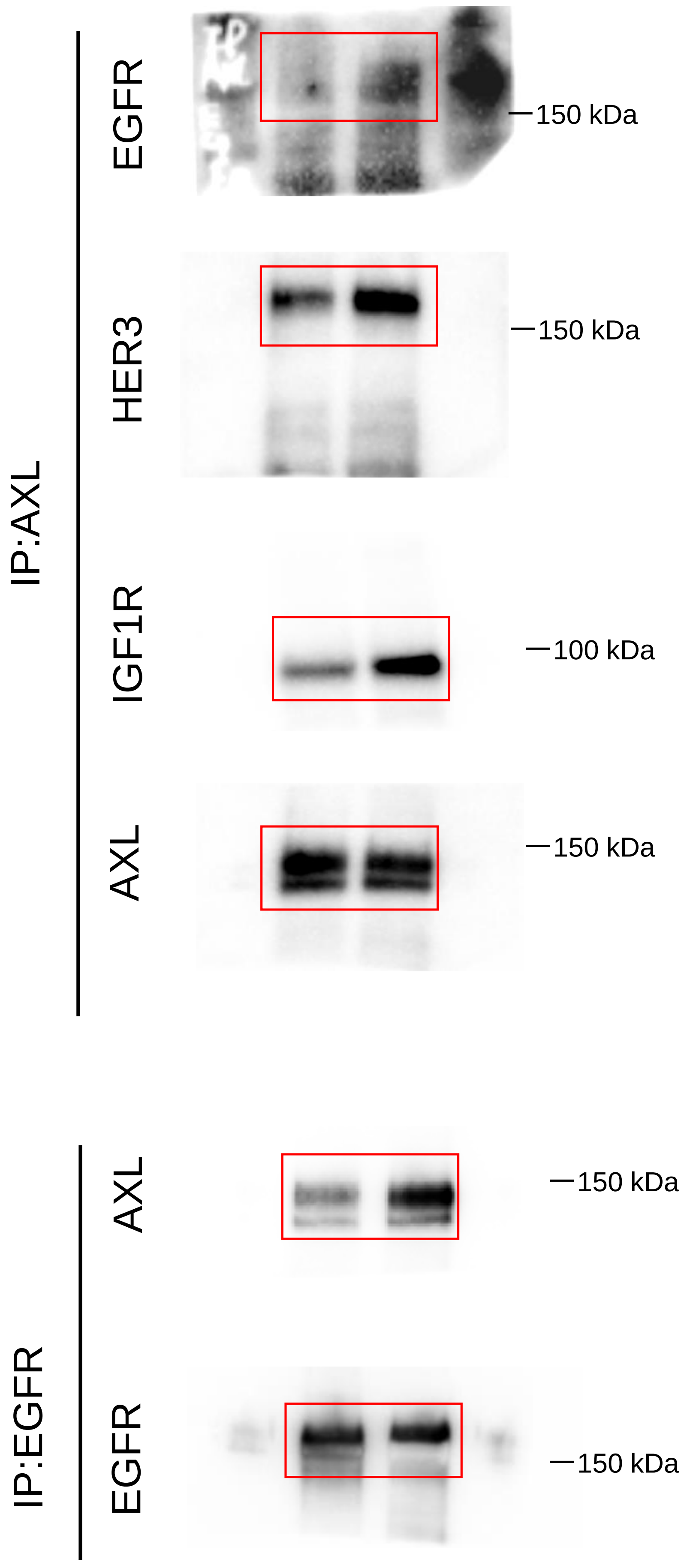

Supplemental Figure 17

Figure. 5E

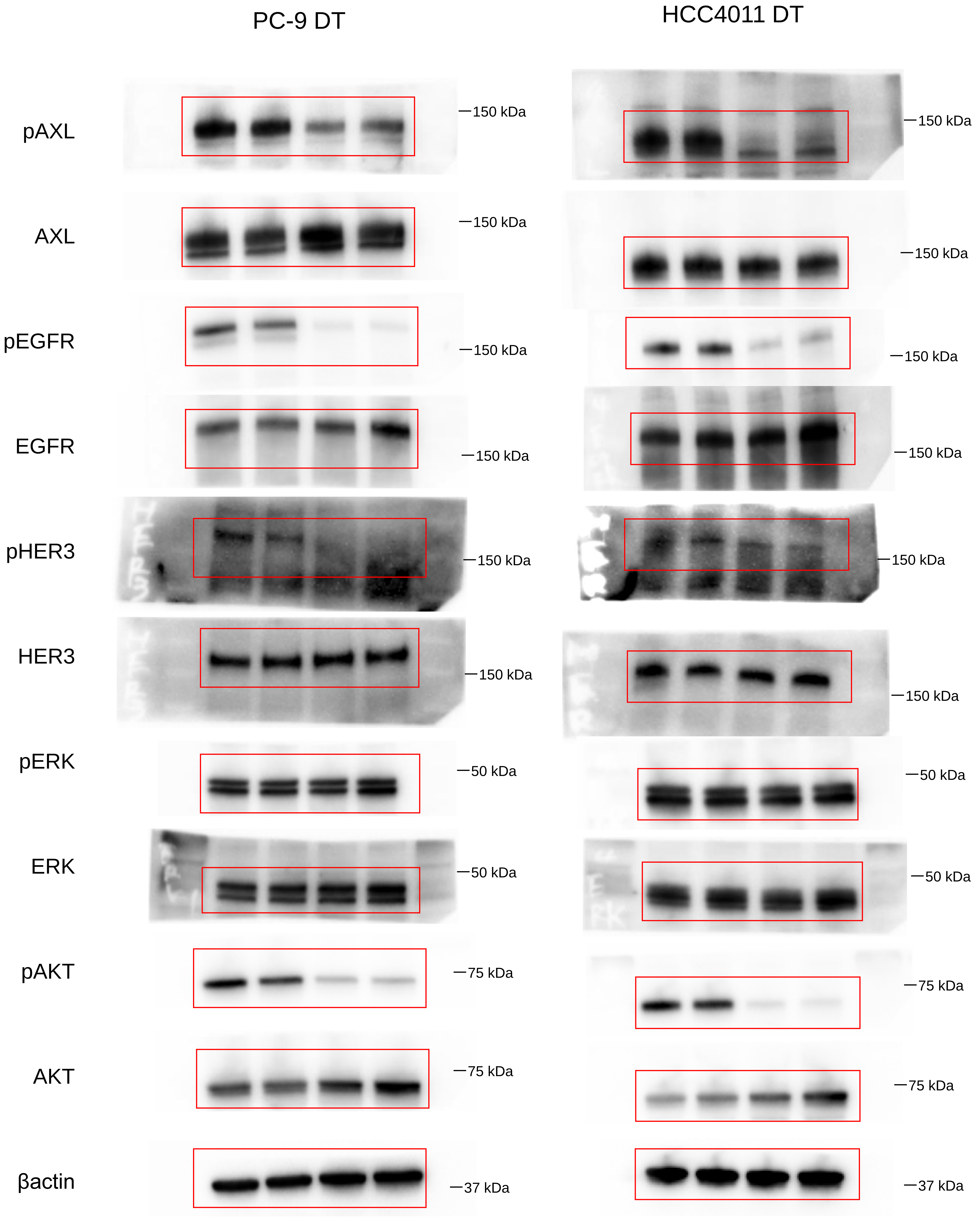

Supplemental Figure 17

Figure. 6A

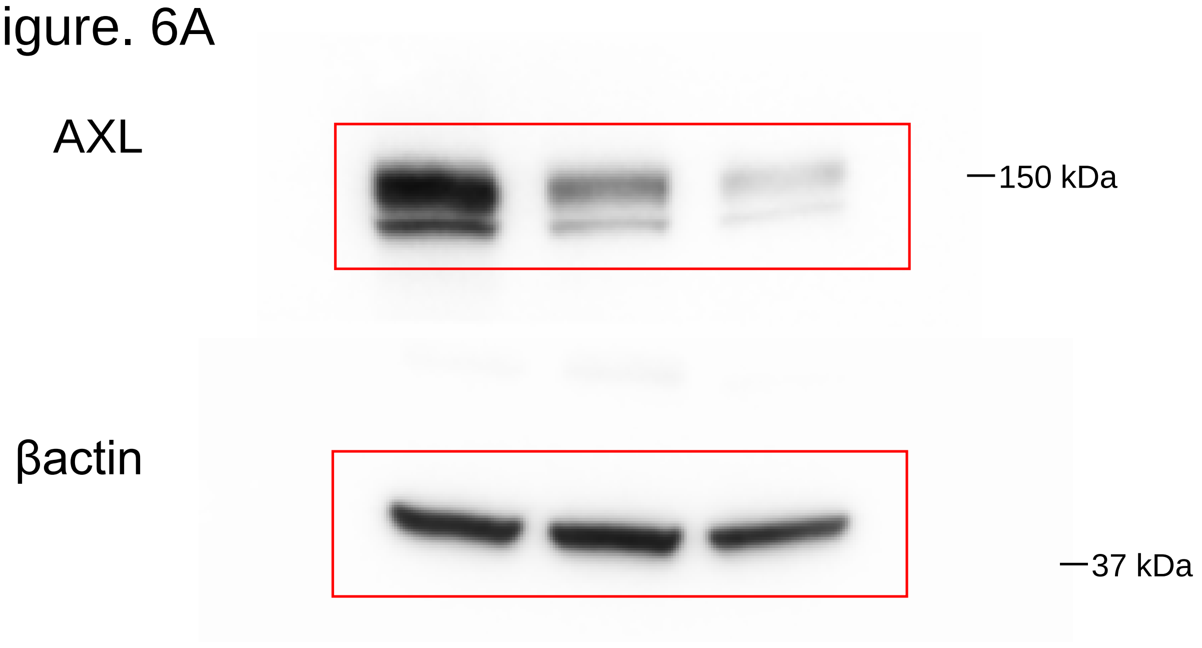

Figure. 6E

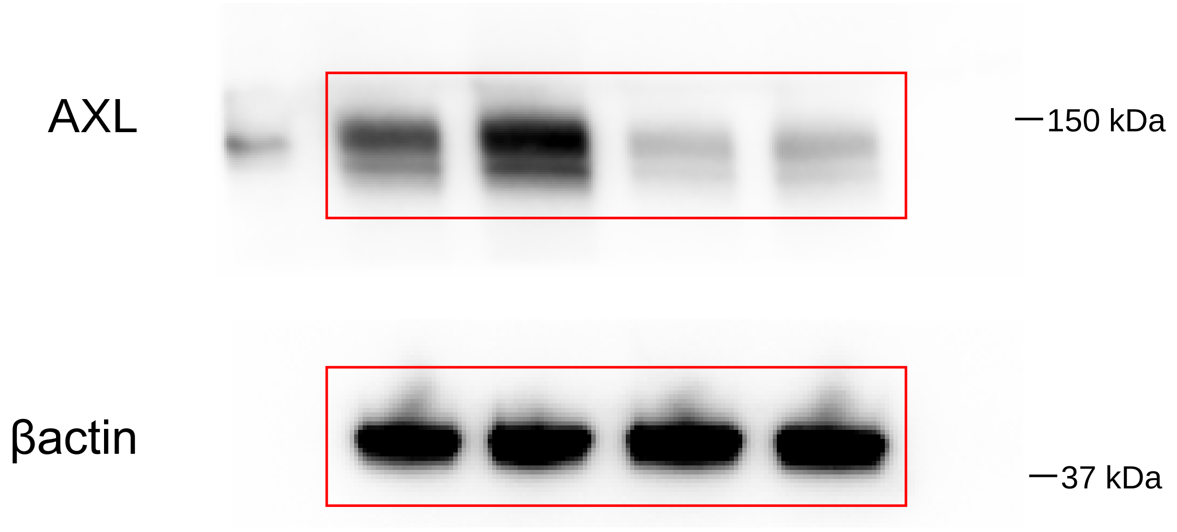

Figure. 7C

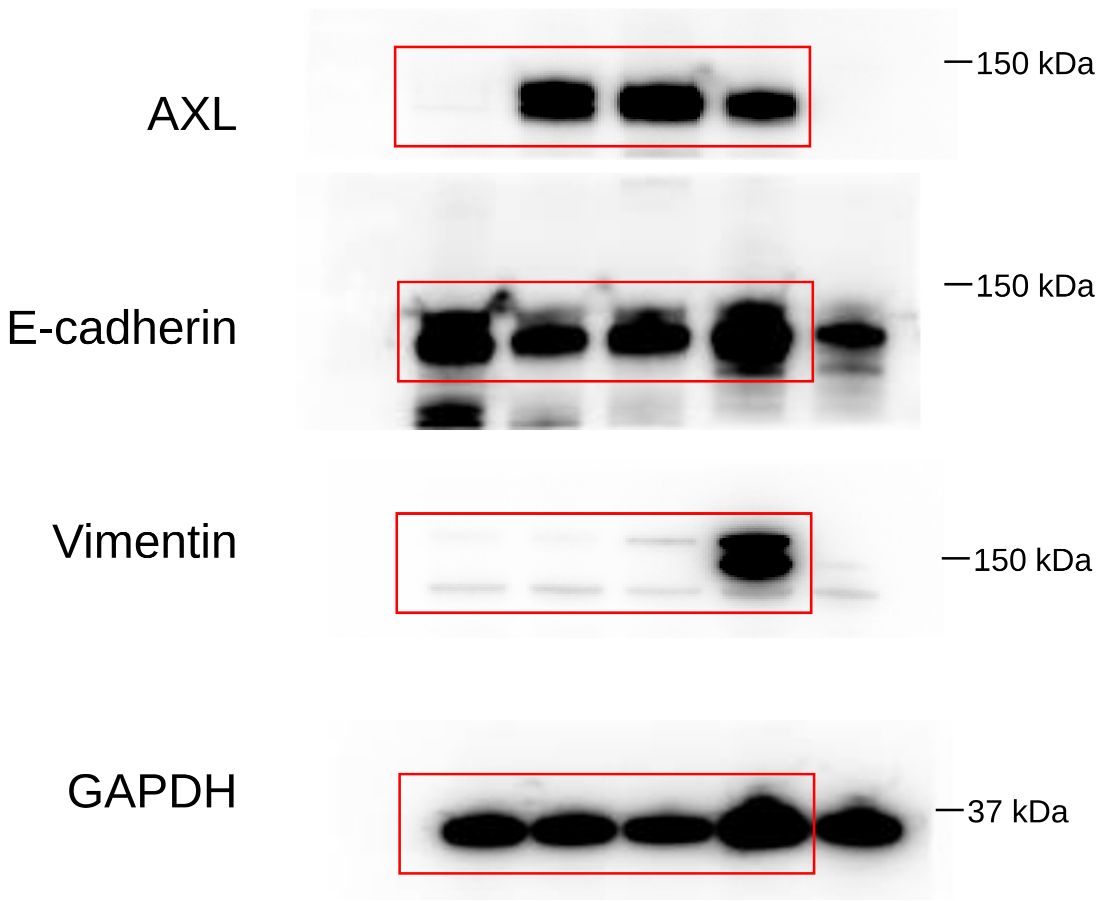

Supplementary Table 1

	Spearman r	p value
AXL/βactin	0.7333	0.0311
pEGFR/βactin	-0.6	0.0968
EGFR/βactin	-0.3167	0.4101
E-Cadherin/βactin	0.5	0.1777
N-Cadherin/βactin	0.4167	0.2696
pHER3/βactin	-0.3667	0.3363
HER3/βactin	-0.08333	0.8432
pERK/βactin	0.2333	0.5517
ERK/βactin	0.5667	0.1206
pAKT/βactin	0.3	0.4366
AKT/βactin	0.2167	0.5809
ALDH1A1/βactin	-0.5333	0.1475
CD44/βactin	0.65	0.0666
SPRY4/βactin	0.05	0.9116

Supplementary Table 1. The correlation between the ratio of indicated protein to βactin and the IC50 values for the osimertinib in EGFR-mutated NSCLC cells by Spearman rank correlation coefficient.

Supplementary Table 2

Cells	EGFR status	Percentage (%)	EGFR Copy Number
PC-9	Wild type	12.56	4.30
	Exon 19 Del	87.46	
PC-9 DT	Wild type	11.81	4.54
	Exon 19 Del	88.19	
HCC4011	Wild type	7.93	10.58
	L858R	92.07	
HCC4011 DT	Wild type	8.54	9.78
	L858R	91.46	

Supplementary Table 1. The status and copy number of the *EGFR* gene in *EGFR*-mutated NSCLC cells and their drug-tolerant (DT) cells.

DNA was extracted from *EGFR*-mutated NSCLC cells (PC-9 and HCC4011) and their DT cells produced by treatment with 3 μmol/L osimertinib for nine days. Their *EGFR* gene status and copy number were evaluated as described in the Methods.

Supplementary Table 3

Sample	Treatment	EGFR C797S mutation
PC-9 shSCR	Control	—
	Osimertinib 2.5mg/kg	—
	Osimertinib 5mg/kg	—
PC-9 shAXL	Control	—
	Osimertinib 2.5mg/kg	—
	Osimertinib 5mg/kg	—

Supplementary Table 2. Analysis of the C797S *EGFR*-mutation present in PC-9 tumors that were generated *in vivo* by the introduction of AXL-targeting and nonspecific shRNAs and osimertinib treatment.
