# Supplementary material for: AXL confers intrinsic resistance to osimertinib and advances the emergence of tolerant cells
Source: Nat Commun. 2019 Jan 16;10:259. doi: 10.1038/s41467-018-08074-0 (PMC6335418; doi:10.1038/s41467-018-08074-0)
Supplement: Supplementary file 3 — Reporting Summary [file 41467_2018_8074_MOESM3_ESM.pdf]

nature research

Corresponding author(s): Seiji Yano, Tadaaki Yamada

Last updated by author(s): Dec 10, 2018

Reporting Summary

Nature Research wishes to improve the reproducibility of the work that we publish. This form provides structure for consistency and transparency in reporting. For further information on Nature Research policies, see Authors & Referees and the Editorial Policy Checklist.

Statistics

For all statistical analyses, confirm that the following items are present in the figure legend, table legend, main text, or Methods section.

n/a	Confirmed	
☐	☒	The exact sample size (*n*) for each experimental group/condition, given as a discrete number and unit of measurement
☐	☒	A statement on whether measurements were taken from distinct samples or whether the same sample was measured repeatedly
☐	☒	The statistical test(s) used AND whether they are one- or two-sided<br>*Only common tests should be described solely by name; describe more complex techniques in the Methods section.*
☐	☒	A description of all covariates tested
☐	☒	A description of any assumptions or corrections, such as tests of normality and adjustment for multiple comparisons
☐	☒	A full description of the statistical parameters including central tendency (e.g. means) or other basic estimates (e.g. regression coefficient) AND variation (e.g. standard deviation) or associated estimates of uncertainty (e.g. confidence intervals)
☐	☒	For null hypothesis testing, the test statistic (e.g. *F*, *t*, *r*) with confidence intervals, effect sizes, degrees of freedom and *P* value noted<br>*Give P values as exact values whenever suitable.*
☒	☐	For Bayesian analysis, information on the choice of priors and Markov chain Monte Carlo settings
☒	☐	For hierarchical and complex designs, identification of the appropriate level for tests and full reporting of outcomes
☒	☐	Estimates of effect sizes (e.g. Cohen's *d*, Pearson's *r*), indicating how they were calculated

Our web collection on statistics for biologists contains articles on many of the points above.

Software and code

Policy information about availability of computer code

Data collection	no software was used.
Data analysis	GraphPad Prism Ver. 6.0 (GraphPad Software, Inc., San Diego, CA, USA)

For manuscripts utilizing custom algorithms or software that are central to the research but not yet described in published literature, software must be made available to editors/reviewers. We strongly encourage code deposition in a community repository (e.g. GitHub). See the Nature Research guidelines for submitting code & software for further information.

Data

Policy information about availability of data

All manuscripts must include a data availability statement. This statement should provide the following information, where applicable:

- Accession codes, unique identifiers, or web links for publicly available datasets
- A list of figures that have associated raw data
- A description of any restrictions on data availability

The authors declare that all data supporting the findings of this study are available within the paper and its supplementary information files.

Field-specific reporting

Please select the one below that is the best fit for your research. If you are not sure, read the appropriate sections before making your selection.

☒ Life sciences ☐ Behavioural & social sciences ☐ Ecological, evolutionary & environmental sciences

For a reference copy of the document with all sections, see nature.com/documents/nr-reporting-summary-flat.pdf

Life sciences study design

All studies must disclose on these points even when the disclosure is negative.

Sample size	This research mainly targets for experimental research.
Data exclusions	Exclusion criteria is none.
Replication	Each experiment was independently performed at least twice.
Randomization	In mice experiments, the mice were transferred to the animal facility at Kanazawa University and randomized once their mean tumor volume reached the each indicated volume.
Blinding	Our mice experiments could not blind to each mice groups.

Reporting for specific materials, systems and methods

We require information from authors about some types of materials, experimental systems and methods used in many studies. Here, indicate whether each material, system or method listed is relevant to your study. If you are not sure if a list item applies to your research, read the appropriate section before selecting a response.

Materials & experimental systems

n/a	Involved in the study	
☐	☒	Antibodies
☐	☒	Eukaryotic cell lines
☒	☐	Palaeontology
☐	☒	Animals and other organisms
☐	☒	Human research participants
☒	☐	Clinical data

Methods

n/a	Involved in the study	
☒	☐	ChIP-seq
☒	☐	Flow cytometry
☒	☐	MRI-based neuroimaging

Antibodies

Antibodies used

p-AXL (Tyr702), t-AXL, p-EGFR, p-MET, t-MET, p-HER3 (Tyr1289), t-HER3, p-IGF-1R, t-IGF-1R, p-Akt (Ser473), t-Akt, E-cadherin, N-cadherin, Vimentin, ALDH1A1, CD44, β-actin (13E5) (Cell Signaling Technology, Danvers, MA, USA), p-Erk1/2 (Thr202/Tyr204), t-Erk1/2, t-EGFR (R&D systems), SPRY4 (Proteintech, Rosemont, IL).

Validation

p-AXL (Tyr702) :Phospho-Axl(Tyr702) （D12B2) Rabbit mAb, #5724
t-AXL: AXL Rabbit mAB, #4566
p-EGFR: Phospho-EGF Receptor(Tyr1068) （D7A5) XP Rabbit mAb, #3777
p-MET: Phospho-Met （Tyr1234/1235)(D26)XP Rabbit mAb, #3077
t-MET: Met （25H2） Mouse mAb, #3127
p-HER3 (Tyr1289) : Phospho-Her3/ErbB3 （Tyr1289)(21D3) Rabbit mAb, #4791
t-HER3: HER3/ErbB3 (1B2E) Rabbit mAb, #4754
p-IGF-1R: Phospho-IGF-I Receptor β (Tyr1131)/Insulin Receptor β (Tyr1146) Antibody, #3021
t-IGF-1R: IGF-I Receptor β (D23H3) XP® Rabbit mAb, #9750
p-Akt (Ser473): Phospho-Akt (Ser473) (D9E) XP® Rabbit mAb, #4060
t-Akt: Akt (pan) (C67E7) Rabbit mAb, #4691
E-cadherin: E-Cadherin(24E10), #3195
N-cadherin:N-Cadherin Rabbit mAb (D4R1H), #13116
Vimentin: Vimentin(D21H3) XP Rabbit mAb, #5741
ALDH1A1: ALDH1A1 (D9Q8E) XP® Rabbit mAb, #54135
CD44: CD44(156-3C11) Mouse mAb, #3570
β-actin: β-Actin(13E5) Rabbit mAb, #4970
p-Erk1/2 (Thr202/Tyr204):anti-phospho-ERK1/ERK2, AF1018
t-Erk1/2: anti-ERK1/ERK2, AF1576
t-EGFR:Anti-hEGFR Antibody, AF231
SPRY4: SPRY4 Rabbit Polyclonal antibody, 22765-1-AP

Eukaryotic cell lines

Policy information about cell lines

Cell line source(s)

HCC4011 and H3255 were generously provided by Drs. David P. Carbone (Ohio State University Comprehensive Cancer Center, Columbus, OH) and John D. Minna (University of Texas Southwestern Medical Center, Dallas, TX), respectively. The H1975 was kindly provided by Drs. Yoshitaka Sekido (Aichi Cancer Center Research Institute, Japan) and John D. Minna. The

	HCC827, HCC4006, and H1650 were purchased from the American Type Culture Collection (Manassas, VA), and the PC-9 cell line was obtained from RIKEN Cell Bank (Ibaraki, Japan). The PC-9KGR cells, which contain deletions in the EGFR exon 19 and the T790M mutation, were developed from PC-9 cells by stepwise exposure to gefitinib. The PC-9GXR cells, which contain deletions in the EGFR exon 19 and the T790M mutation, were established at Kanazawa University (Kanazawa, Japan) from PC-9 cell xenograft tumors in nude mice that had acquired resistance to gefitinib.
Authentication	Cell lines were authenticated by DNA fingerprinting.
Mycoplasma contamination	Cells were regularly screened for mycoplasma using a MycoAlert Mycoplasma Detection Kit.
Commonly misidentified lines (See ICLAC register)	Commonly misidentified lines were not used in the study.

Animals and other organisms

Policy information about studies involving animals; ARRIVE guidelines recommended for reporting animal research

Laboratory animals	Five-week-old male mice with severe combined immunodeficiency (SCID) obtained from Clea Japan (Tokyo, Japan). Female NOD.Cg-Prkdcscid Il2rgtm1Wjl/SzJ (NSG) mice aged 6–8 weeks were engrafted with tumor fragments at passage P7 at The Jackson Laboratory.
Wild animals	The study did not include wild animals.
Field-collected samples	The study did not involve samples collected from the field.
Ethics oversight	The study protocol was approved by the Ethics Committee on the Use of Laboratory Animals and the Advanced Science Research Center, Kanazawa University, Kanazawa, Japan (approval no. AP-122505).

Note that full information on the approval of the study protocol must also be provided in the manuscript.

Human research participants

Policy information about studies involving human research participants

Population characteristics	Specimens of tumors containing EGFR-activating mutations prior to the initial treatments with EGFR-TKIs were obtained from 46 lung adenocarcinoma patients hospitalized at Kanazawa University Hospital (Kanazawa, Japan), Niigata University Hospital (Niigata, Japan), Niigata Cancer Center Hospital (Niigata, Japan), Nagasaki University Hospital (Nagasaki, Japan), or the Japanese Red Cross Nagasaki Genbaku Hospital (Nagasaki, Japan). Specimens of tumors containing EGFR-activating mutations, prior to treatment with osimertinib, were obtained from 11 lung adenocarcinoma patients hospitalized at the Kanazawa University Hospital (Kanazawa, Japan), Japanese Red Cross Kyoto Daiichi Hospital (Kyoto, Japan), Niigata University Hospital (Niigata, Japan), and Niigata Cancer Center Hospital (Niigata, Japan).
Recruitment	All patients were participants in Institutional Review Board of each Hospitals –approved studies.
Ethics oversight	All patients were participants in Institutional Review Board of each Hospital –approved studies and all provided written informed consent.

Note that full information on the approval of the study protocol must also be provided in the manuscript.
